# Unraveling abiotic organic synthesis pathways in the mafic crust of mid-ocean ridges

**DOI:** 10.1073/pnas.2308684121

**Published:** 2024-10-10

**Authors:** Jingbo Nan, Xiaotong Peng, Oliver Plümper, Iris C. ten Have, Jing-Guang Lu, Qian-Bao Liu, Shao-Lin Li, Yingjie Hu, Yu Liu, Zhen Shen, Weiqi Yao, Renbiao Tao, Martina Preiner, Yongxiang Luo

**Affiliations:** ^a^Institute of Deep-Sea Science and Engineering, Chinese Academy of Sciences, Sanya 572000, China; ^b^Nanjing Institute of Geology and Palaeontology, Chinese Academy of Sciences, Nanjing 210008, China; ^c^Center for High Pressure Science and Technology Advanced Research, Beijing 100094, China; ^d^Department of Earth Sciences, Faculty of Geosciences, Utrecht University, Utrecht 3584 CD, The Netherlands; ^e^Debye Institute for Nanomaterials Science, Faculty of Science, Utrecht University, Utrecht 3584 CG, The Netherlands; ^f^State Key Laboratory of Quality Research in Chinese Medicines, Macau Institute for Applied Research in Medicine and Health, Macau University of Science and Technology, Taipa, Macau 999078, China; ^g^The State Key Laboratory of Lunar and Planetary Science, Macau University of Science and Technology, Taipa, Macau 999078, China; ^h^Nanjing Key Laboratory of Advanced Functional Materials, Nanjing Xiaozhuang University, Nanjing 211171, China; ^i^College of Geoscience and Surveying Engineering, China University of Mining and Technology (Beijing), Beijing 100083, China; ^j^Department of Ocean Science and Engineering, Southern University of Science and Technology, Shenzhen 518055, China; ^k^Microcosm Earth Center, Max Planck Institute for Terrestrial Microbiology and Philipps Universität Marburg, Marburg 35032, Germany; ^l^Center for Synthetic Microbiology, Marburg 35032, Germany; ^m^Geochemical Protoenzymes Research Group, Max Planck Institute for Terrestrial Microbiology, Marburg 35043, Germany

**Keywords:** low-temperature alteration of oceanic crust, abiotic organic synthesis, organic condensation, mineral-catalyzed reaction, deep carbon cycle

## Abstract

The aqueous alteration of the oceanic lithosphere provides significant energy that impacts the synthesis and diversity of organic compounds, which are crucial for the deep carbon cycle and may have provided the first building blocks for life. Although abiotic organic synthesis has been documented in mantle-derived rocks, the formation mechanisms and complexity of organic compounds in crustal rocks remain largely unknown. Here, we show the specific association of aliphatic carbonaceous matter with Fe oxyhydroxides in mafic crustal rocks of the Southwest Indian Ridge (SWIR). We determine potential Fe-based pathways for abiotic organic synthesis from CO_2_ and H_2_ using multimodal and molecular nano-geochemical tools. Quantum mechanical modeling is further employed to constrain the catalytical activity of Fe oxyhydroxides, revealing that the catalytic cycle of hydrogen may play a key role in carbon–carbon bond formation. This approach offers the possibility of interpreting physicochemical organic formation and condensation mechanisms at an atomic scale. The findings expand our knowledge of the existence of abiotic organic carbon in the oceanic crustal rocks and emphasize the mafic oceanic crust of the SWIR as a potential site for low-temperature abiotic organic synthesis.

## Significance

This study explores the mechanism of abiotic organic synthesis in the oceanic crust, a process crucial for deep carbon cycle, deep biosphere, and potentially life's origin. We reveal a unique link between carbonaceous matter and iron-based compounds in mafic crustal rocks at the Southwest Indian Ridge (SWIR). Using nano-geochemical tools and quantum mechanical modeling, we uncover potential pathways for nonbiological organic synthesis from CO_2_ and H_2_, emphasizing the catalytic cycle of hydrogen in carbon–carbon bond formation. Having identified abiotic organic compounds in specific mafic rock clasts, our study indicates possible avenues for nonbiological processes in analogous formations. Consequently, the mafic oceanic crust of the SWIR emerges as a prospective area for delving deeper into low-temperature abiotic organic synthesis.

The interaction of hydrothermal fluids with the oceanic lithosphere results in a plethora of geochemical changes that not only affect geological processes but also impact the existing deep biosphere (1). Within this context, the reaction of fluids with (ultra)mafic igneous and metamorphic rocks may generate copious amounts of hydrogen (H_2_) (2, 3). This hydrogen then interacts with catalytically active minerals in the presence of a carbon source (e.g., CO_2_), aiding the abiotic synthesis of organic molecules (4, 5). These processes are thermodynamically favored in hydrothermal environments (6–8) and may not be limited to the hydration of mantle rocks (serpentinization) in the deep lithosphere. Instead, they can also be prevalent in the shallower mafic crust, where ascending fluids from the deep lithosphere via serpentinization (<400 °C) (9) and/or in situ low-temperature (<100 °C) alteration collectively provide significant amounts of H_2_ (10). This process also leads to the formation of Fe (oxyhydr)oxides (10–12) with catalytic potential (6, 13). As such, numerous natural observations (14, 15) and experiments (16, 17) suggest that organic compounds are synthesized on the surface of metal-bearing minerals (e.g., saponite, magnetite) or in fluid inclusions (18, 19) within the deep lithosphere, but little is known about potential organic formation pathways in the mafic oceanic crust.

Assessing the origin of rock-hosted organic compounds is challenging (20). Although abiotic organic synthesis has been proposed to occur during oceanic crustal alteration (6–8), it remains difficult to discern these organics from their biological counterparts (21). Previous research reports abiotic methane and disordered graphitic carbon inclusions within the oceanic crust (22). These organic compounds may originate from carbon respeciation during the cooling of CO_2_-rich magmatic gas (22, 23). Recent studies have combined microstructural and microchemical imaging techniques with in situ molecular information to overcome the impasse of determining the origin of organic matter. The intimate association of specific minerals with condensed carbonaceous matter (CCM) provides an insight into the origin of the organics (14, 20, 24). Specifically, Ménez et al. investigated altered mantle rocks along the Mid-Atlantic Ridge, revealing the low-temperature abiotic formation of aromatic amino acids via Friedel-Crafts reactions catalyzed by surrounding iron-rich clays (15). Moreover, Sforna et al. proposed that the formation of abiotic CCM at low temperatures is primarily driven by redox conditions during hydrothermal alteration, particularly in association with hydrogarnet or hematite (Fe_2_O_3_) formation (24). While these pioneering studies have elucidated mechanisms of abiotic organic synthesis, the detailed physicochemical processes underlying these transformations require further exploration to be fully understood.

Here, we use correlative microanalytical tools to investigate the molecular geochemistry of CCM in oceanic basalts from the Southwest Indian Ridge (SWIR), where active hydrothermal circulation is present. Based on the specific association of CCM with Fe oxyhydroxides and the molecular signature of the organic carbon, we determine the potential Fe-based pathway for abiotic organic synthesis during low-temperature alteration of mafic oceanic crust (<150 °C) (25). In this process, nitrogen-bearing compounds, crucial intermediates for generating functional biomolecules, may also form. We further employ quantum mechanical simulations to constrain the catalytic activity of Fe oxyhydroxides in the hydrogenation of CO_2,_ and to assess their role in promoting C-C chain growth and potential condensation mechanisms.

## Results and Discussion

### Mafic Rocks and Fe Oxyhydroxides.

**Fig. 1.**
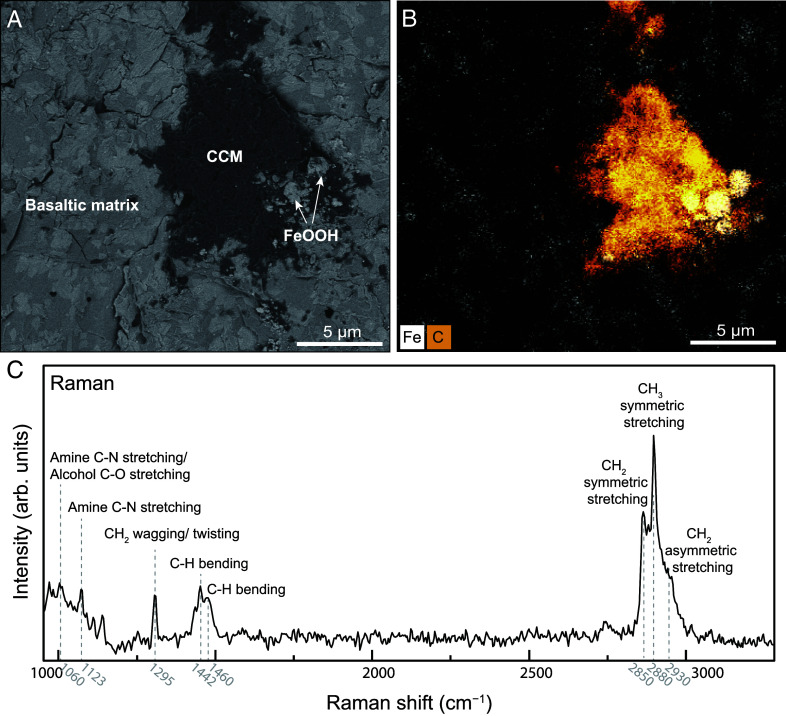
Association of CCM with Fe oxyhydroxides in sample SY122-G06. (*A*) BSE image of the CCM on the unpolished sample surface, and their related Fe oxyhydroxides formed as a result of the oxidation of Fe(II)-bearing minerals in the basaltic matrix. (*B*) Corresponding energy-dispersive X-ray spectrometry map of (*A*), showing the elemental distributions of carbon (orange) and iron (white). (*C*) Raman spectrum of CCM in (*A*). (arb.–arbitrary.)

We studied seven crustal rocks recovered from the SWIR at 2,633 to 2,811 m below sea level (*SI Appendix*, Fig. S1 and Table S1). Notably, the samples we obtained are relatively fresh basalts (*SI Appendix*, Fig. S2) and only display a localized alteration in the matrix (*SI Appendix*, Figs. S3 and S4). The basalts are primarily composed of silicate minerals, including pyroxene, olivine, and plagioclase, which vary in proportion across different samples (*SI Appendix*, Table S1). Silicate phenocrysts constitute about 20 vol.% of the basalts and are embedded in an aphanitic (fine-grained) basaltic matrix with accessory minerals of Fe–Ti oxides and sulfides (*SI Appendix*, Fig. S5). While the basalts exhibit a speckled texture, the matrix is composed of a microcrystalline mixture of pyroxene, olivine, and plagioclase grains (*SI Appendix*, Fig. S6). Specifically, variable amounts of magmatic pore space are present in the form of µm-sized cavities between interstitial minerals and large vesicles (several hundred µm; *SI Appendix*, Figs. S5 and S7). Among the seven basalts, SY122-G06 and SY089-G02 exhibit larger volume fractions of pores and a higher degree of interconnected fractures compared to the others (*SI Appendix*, Fig. S7 and Table S1). This indicates that SY122-G06 and SY089-G02 have enhanced porosity and permeability. Furthermore, the pore space in basalts likely facilitates fluid-driven alteration processes, as evidenced by the presence of Fe oxyhydroxides (*SI Appendix*, Fig. S3). These Fe oxyhydroxides, including goethite (α-FeOOH), akaganeite (β-FeOOH), and lepidocrocite (γ-FeOOH), are widely distributed as rosette-like aggregates (<2 µm) (Fig. 1 and *SI Appendix*, Fig. S3). They are spatially associated with matrix minerals (*SI Appendix*, Fig. S4), and constitute about 2 vol.% of the matrix-based on microscope observations (*SI Appendix*, Table S1). It is interesting to note that the alkali-rich secondary hydrous phyllosilicates are absent in the studied fresh basalts. This absence may be attributed to the rapid precipitation of Fe oxyhydroxides during oxidative alteration (weathering) processes (11, 26, 27), combined with the high mobility and subsequent leaching of alkali elements (28, 29). Additionally, the ascent of Fe-rich but alkali-poor hydrothermal fluids from the deep lithosphere to shallower crustal levels along the SWIR (30) may further explain the exclusive presence of Fe oxyhydroxides without hydrous phyllosilicates in these basalts (also see details in *SI Appendix*).

### Rock-Hosted Organic Matter.

We have identified CCM in two basalts (SY122-G06 and SY089-G02) from the Dragon Horn area and the Tian Cheng area, respectively (*SI Appendix*, Table S1). The CCM is of varying sizes within matrix-based cavities (<50 µm) in direct association with aggregates of Fe oxyhydroxide (Fig. 1 and *SI Appendix*, Figs. S4 and S8). Raman spectroscopy reveals a consistent composition across all investigated CCM in terms of the types of bonds and functional groups present. However, variations in aliphaticity are evident, as demonstrated by differing intensities of CH_2_ bands relative to CH_3_ bands (*SI Appendix*, Fig. S9). Aliphatic compounds are recognized (Fig. 1), with vibrational modes attributable to amine C-N stretching/ alcohol C-O stretching (1,060 cm^−1^), amine C-N stretching (1,123 cm^−1^), aliphatic CH_2_ wagging/twisting (1,300 cm^−1^), and C-H bending (1,450 cm^−1^, 1,460 cm^−1^). Additionally, CH_2_ stretching modes are observed at 2,850 cm^−1^, 2,930 cm^−1^, and CH_3_ stretching at 2,880 cm^−1^. Vibrational bands associated with aromatic moieties were not detected (i.e., absence of bands in the range of 1,500 to 1,600 cm^−1^ and 3,000 to 3,100 cm^−1^). Furthermore, if nitrogen heterocyclic compounds like pyridine (C_5_H_5_N) were present, aromatic C-H stretching vibrations at 3,000 to 3,100 cm^−1^ would be expected. The absence of peaks in this region suggests that such nitrogen heterocyclic compounds are not present in the CCM.

**Fig. 2.**
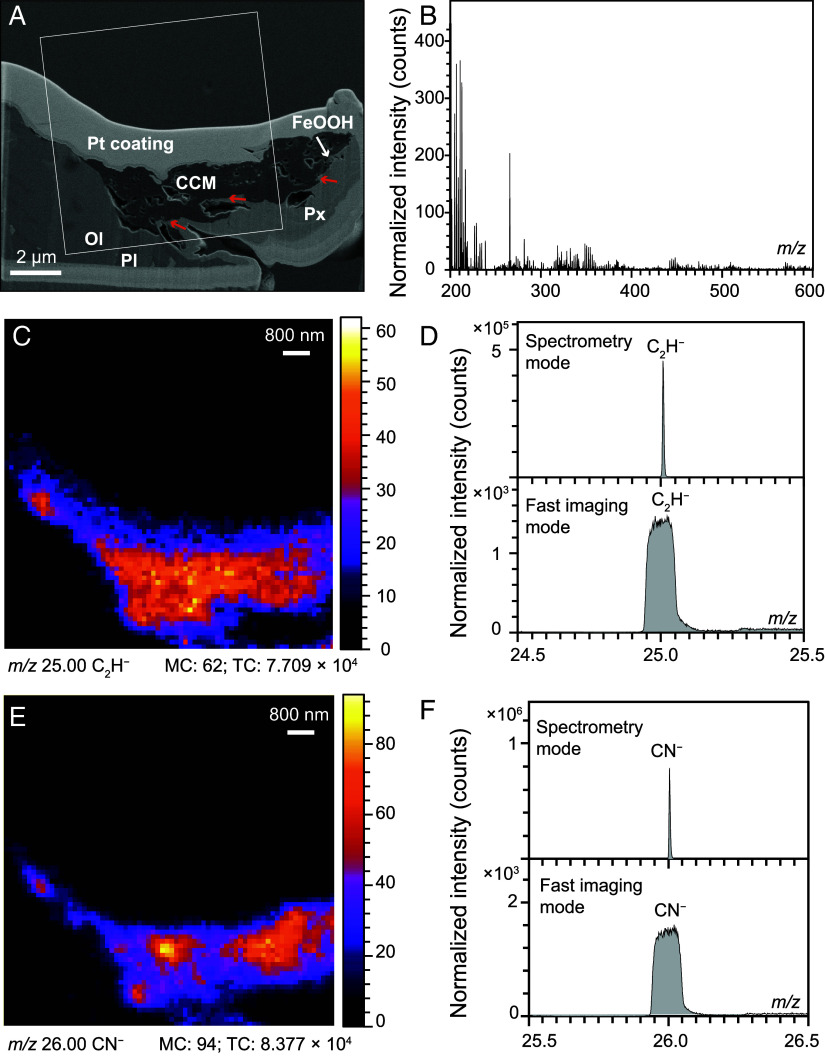
The presence of aliphatic compounds in the CCM from sample SY122-G06. (*A*) BSE image of the FIB-SEM foil (see *SI Appendix*, Fig. S4 for more details); the white box indicates the TOF-SIMS analysis location; the orange arrows indicate the penetration of matrix minerals into the CCM. Ol-olivine, Px-pyroxene, Pl-plagioclase. (*B*) TOF-SIMS spectrum (*m/z* of 200 to 600) reconstructed from the CCM region in (*A*), the spectra were normalized by the total primary dose (*SI Appendix*, Fig. S11). (*C*–*F*) TOF-SIMS imaging shows the distribution of alkyl and amine functional groups, which are characterized by C_2_H^−^ (at 25.0 *m/z*) and CN^−^ (at 26.0 *m/z*) separately.

Time-of-flight secondary ion mass spectrometry (TOF-SIMS) collected from an ultrathin foil prepared via focused ion beam scanning electron microscopy (FIB-SEM) confirms the presence of aliphatic compounds (Fig. 2). More specifically, the TOF-SIMS mass spectrum is correlated to aliphatic fragment ion species (e.g., C_2_H^−^, C_2_OH^−^, CN^−^), characteristic of alkyl, oxygenated, and nitrogen-containing functional groups (Fig. 2). Additional fragment ions are also consistent with the structure of aliphatic compounds (*SI Appendix*, Fig. S10). It is noteworthy that the CCM exhibits a heterogeneous distribution of nitrogen-containing functional groups (Fig. 2*E*). Common biomarkers from marine-dissolved organic carbon or microbial life, such as pristane, squalene, lycopane, were not detected (31–33) (*SI Appendix*, Fig. S11). Given the high sensitivity of TOF-SIMS, the lack of biomarker signals indicates either the absence of biological compounds within CCM or their significantly lower contents compared to abiotic counterparts.

**Fig. 3.**
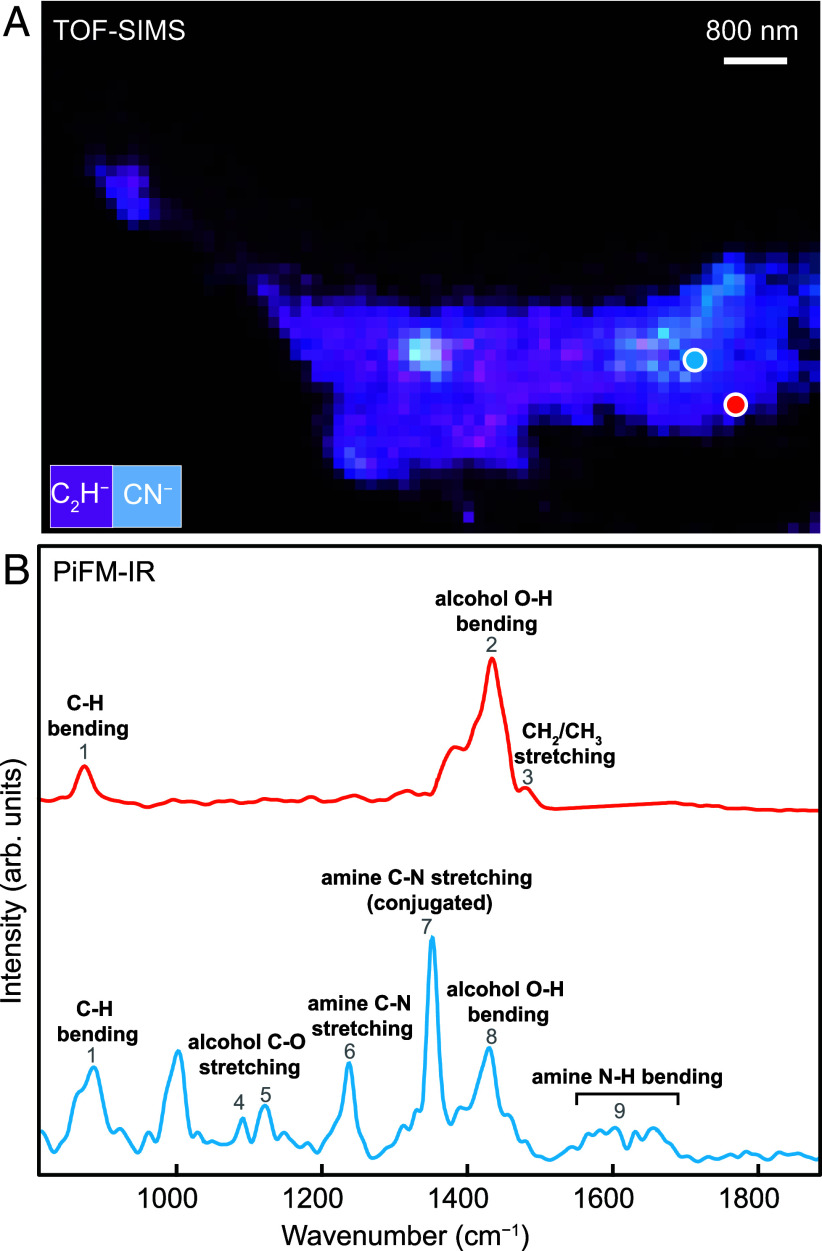
Nanoscale molecular information of the CCM. (*A*) TOF-SIMS mapping showing association of C-H (purple) and CN (blue) in CCM (corresponds to Fig. 2). (*B*) PiFM-IR spectra collected in (*A*). The colors of the position marker correspond to the colored spectra. Red and blue color spectra depict alkyl and amine functional groups, respectively, and also indicate the presence of alcohol functional groups. See also *SI Appendix*, Table S2.

We have also employed photoinduced force microscopy (PiFM) to investigate the submicron-scale molecular character of the CCM identified on the same FIB foil. PiFM allows us to collect infrared spectra with a sub-20 nm spatial resolution. The molecular information obtained by PiFM is consistent with Raman and TOF-SIMS analysis, further confirming the presence of aliphatic moieties (Fig. 3*B*). Specifically, the obtained PiFM spectra highlight the presence of alkyl (at 880 cm^−1^, 1,462/1,472 cm^−1^), alcohols (at 1,085 cm^−1^, 1,115 cm^−1^, 1,420 to 1,422 cm^−1^), and amine functional groups (at 1,230 cm^−1^, 1,344 cm^−1^, 1,580 to 1,650 cm^−1^). No amides and carboxyl acids were detected, given the absence of bands at 1,680 to 1,690 cm^−1^ and 1,700 to 1,750 cm^−1^ attributable to C=O vibration. The absence of peaks in the region corresponding to aromatic C-H stretching vibrations and overtones (1,750 to 2,080 cm^−1^) also indicates that nitrogen heterocyclic compounds are not present in the CCM.

### Assessment of Sample Contamination and Exogenous Organics from Seawater.

The samples collected by the HOV were immediately frozen at −20 °C once on board. To limit any organic contamination after seafloor sampling, centimeter-sized subsamples were extracted from the inner regions of the original sample. All subsamples were cut using sterile ultrapure water or freshly opened in a mortar with a pestle. No epoxy and polishing procedures were introduced to limit the contact of the sample surface with potential organic-bearing materials. Organic-lean blank samples were processed within the same approach as all other samples. In addition, the saw and mortar were pretreated with pure ethanol and rinsed with sterile ultrapure water throughout the procedure. Before analysis, all subsample surfaces were cleared of dust using high-speed, ultrapure (>99.9%) nitrogen gas. To investigate any potential contamination during rock cutting, we compared the Raman spectra of CCM from cut and pestled subsamples (*SI Appendix*, Fig. S9). The relatively consistent spectra across all investigated CCM suggest that the CCM within cut samples was not derived from laboratory contamination. In addition, if the CCM was introduced in subsequent steps after seafloor sampling, the organics would be distributed randomly throughout the sample instead of showing a direct association with Fe oxyhydroxides. The presence of CCM within inner cavities (24, 34) instead of only on the sample surface, and the penetration of matrix minerals into the CCM (14) (Fig. 2*A*) further exclude the possibility of contamination during sample preparation.

The modern oceanic seafloor basement is affected by long-lived seawater alteration. Exogenous biological contamination of present-day microbial ecosystems may have been introduced via seawater infiltration overprinting any endogenic organic signatures originally formed within the rock. In this case, a direct relationship of the CCM with fracture networks as the primary fluid pathways would be expected. However, such a relationship is absent (*SI Appendix*, Fig. S5) as CCM is exclusively found associated with Fe oxyhydroxides that are heterogeneously distributed throughout rock samples. More importantly, background organic contamination in modern seawater mainly consists of biological compounds. The absence of highly refractory biomarkers, such as pristane, cholestane, squalene, lycopane, β-carotene, and hopanoids, confirmed by TOF-SIMS (31–33) (*SI Appendix*, Fig. S11) further excludes the possible presence of seawater-derived biological contamination. Hence, although the rock studied here may have been subjected to seawater circulation, structural associations imply that the CCM reported here derived from endogenic processes within the mafic oceanic crust.

### Abiotic Genesis of the Organic Carbon.

Two possibilities remain that may explain the presence of endogenic organic carbon within the samples investigated here. Either the CCM reported here results from in situ microbial activity or it is of abiotic origin. With respect to the former, previous studies suggested that large vesicles or cracks within the oceanic crust provide ideal space for the migration and colonization of microorganisms that have filamentous, microfossil-like structures (35, 36). Here, in contrast, CCM is consistently found within micropores that lack filaments and is directly associated with Fe oxyhydroxides (Fig. 1 and *SI Appendix*, Figs. S4 and S8). Although microfossil structures can be destroyed by thermal degradation, leaving remnants of organic matter (34, 37), TOF-SIMS results further suggest that the molecular character of the CCM differs distinctly from that found in microbial cells and extracellular polymeric substances (*SI Appendix*, Fig. S11). Hence, we suggest that the CCM is not of microbial origin.

As Fe oxyhydroxides are well-known catalysts for the organic synthesis of linear hydrocarbons (13, 38) and given the intimate relationship to the CCM identified here, we suggest that the Fe oxyhydroxide within the altered basalt has acted as a catalyst for the synthesis of the organics. During subseafloor alteration, mixing of hydrothermal fluids and cold, oxygenated seawater results in disequilibrium environments (39), which allow rapid crystallization and repeated Fe oxyhydroxide nucleation, leading to the formation of small crystals with high surface areas. This crystal architecture may lead to enhanced adsorption capacities for molecules and catalytic activity (13, 40).

The aqueous alteration of the oceanic lithosphere generates significant energy, during which H_2_ is produced via serpentinization of deep mantle rocks and low-temperature alteration of the shallower mafic crust. Tao et al. investigated the geochemical composition of the hydrothermal fluids in the Dragon Horn area, which exhibited H_2_ concentrations of around 0.3 mM. Their study on vent fluid compositions suggests that both mafic and ultramafic rocks are involved in the larger hydrothermal systems (30). Moreover, Gallant and von Damm have detected CO_2_ within hydrothermal systems along the Indian Ridge akin to the Dragon Horn area (41). Hence, CO_2_-bearing fluids accompanied by mafic-ultramafic alteration-generated H_2_ at Dragon Horn could provide an initial source for abiotic organic synthesis (42). Indeed, H_2_ concentrations below 0.7 mM, similar to those at the Dragon Horn area, have been noted in the mafic-hosted hydrothermal vents of Menez Gwen at the Mid-Atlantic Ridge, potentially contributing to the abiotic synthesis of CH_4_ (18). Furthermore, geochemical evidence suggests nitrogen (N_2_ and NH_3_) degassing at mid-ocean ridges (43), where hydrothermal fluids contain high nitrogen concentrations (4.5 to 6.1 mM NH_4_^+^) (44) and ammonia-oxidizing microbes flourish (45). While the reaction pathways for the abiotic formation of amine functional groups under hydrothermal conditions require further investigation, the amination of alcohols using ammonia or cyanide is one of the most commonly cited reactions in industrial contexts (46, 47). This overall reaction can be favored under aqueous conditions at temperatures below 200 °C (47, 48) and may be catalyzed by iron-based catalysts (46). Therefore, the abiotic amine functional groups observed in the CCM (Fig. 3) may have been formed through amination reactions catalyzed by Fe oxyhydroxides. During this process, the discharge of nitrogen-bearing fluids, combined with the synthesized abiotic alcohol functional groups, likely served as reactants. This may also explain the heterogeneous distribution of nitrogen-containing functional groups in the CCM (Fig. 2*E*). Additionally, the presence of CCM is only detected in samples SY122-G06 and SY089-G02, which exhibit the highest volume fractions of vesicles and interconnected fractures (*SI Appendix*, Fig. S7 and Table S1). This correlation suggests that the enhanced porosity and permeability in SY122-G06 and SY089-G02 may facilitate more extensive fluid circulation, containing H_2_, CO_2_, and nitrogen-rich fluids, thereby promoting the potential for CCM formation.

### Mechanistic Constraints on the Formation of Condensed Organic Compounds.

To gain a comprehensive understanding of abiotic organic synthesis, it is important to study both its thermodynamic and kinetic aspects. Previous studies have effectively employed thermodynamic calculations to understand abiotic organic synthesis in hydrothermal systems (5, 8, 15). Andreani and Ménez have discussed the different stages of abiotic carbon in a heterogeneous oceanic lithosphere based on natural observations, experiments, and thermodynamic calculations (20). They suggest that the strongest thermodynamic drive for metastable organic molecules, including *n*-alkanes, is for typical redox conditions occurring in the mafic oceanic crust at low-temperature conditions. This aligns with our observation of alkylated CCM in the mafic rock of the SWIR. Further, thermodynamic calculations show the chemical imbalances created when a variety of hydrothermal fluids derived from mafic and ultramafic rocks mix with seawater (5). Quantifying the chemical affinity between equilibrium and mixed compositions highlights reactions with a strong thermodynamic drive required for abiotic organic synthesis. These reactions demonstrate notably high chemical affinities for *n*-alkanes and *n*-alcohols, particularly at low temperatures. This indicates that the organic synthesis of these compounds is thermodynamically favorable in low-temperature hydrothermal systems.

**Fig. 4.**
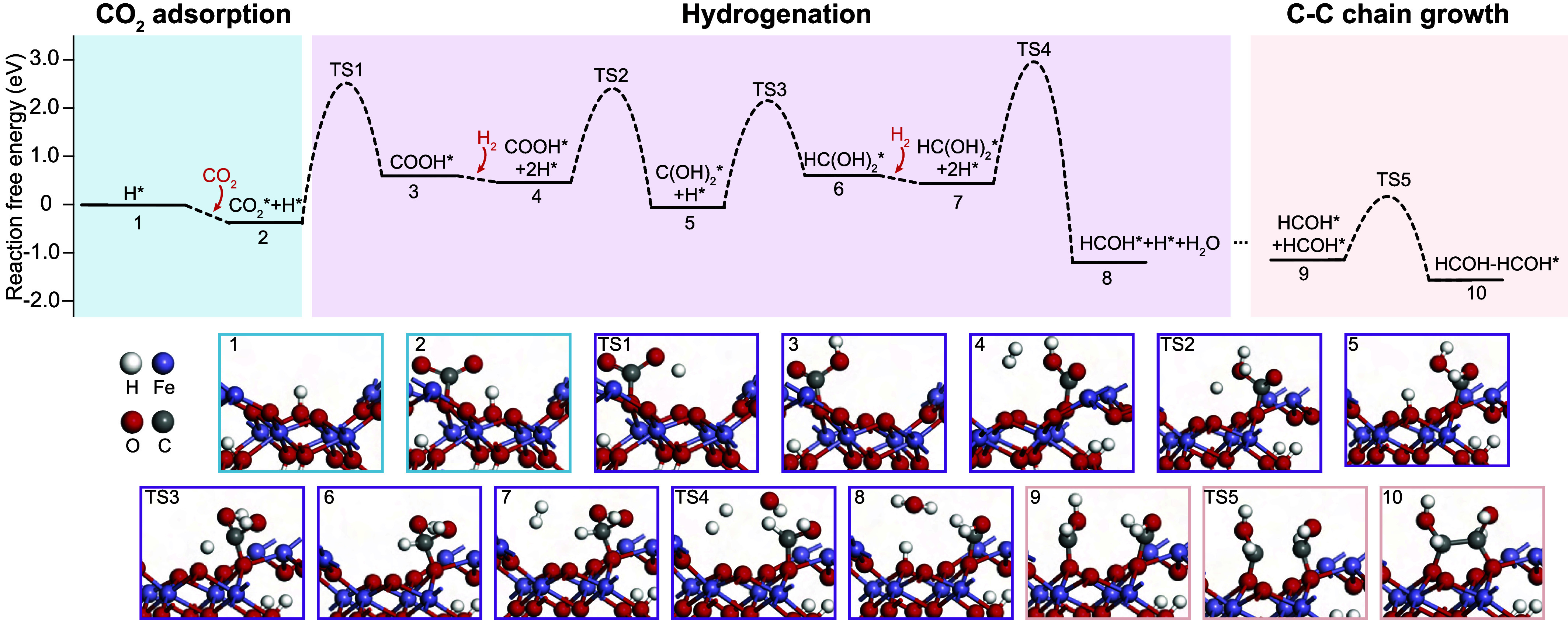
Quantum mechanical modeling with free-energy diagram of the reaction pathways, illustrating CO_2_ hydrogenation and C-C chain growth on the α-FeOOH (001) surface. Optimized structures of each state associated with the hydrogenation process are included. (TS—transition state.)

Although thermodynamic constraints on abiotic organic production have been well established, the role of mineral phases in terms of kinetics and pathway selectivity of natural synthesis still requires further elucidation. In this study, to determine the catalytic potential of Fe oxyhydroxides in organic synthesis and to understand the kinetic condensation mechanism, we performed density functional theory (DFT) simulations (Fig. 4). This method can provide a theoretical framework to analyze and predict the behavior of hydrogenation reactions at a molecular level (49).

As a first step, we investigated the adsorption of a CO_2_ molecule onto a (001) goethite (α-FeOOH) surface (state 1). After structural optimization, we found that CO_2_ interacts with the Fe oxyhydroxide surface, where the C atom is directly bonded to the O atom and forms a carbonate-shaped group (state 2), in agreement with previous investigations (50). The adsorption of CO_2_ to the surface is exothermic (−0.40 eV), indicating that goethite can effectively capture CO_2_. Subsequent CO_2_ dissociation (CO_2_* → CO* + O*) on Fe (oxyhydr)oxides is typically hindered by a high kinetic barrier (51). Instead, adsorbed CO_2_ (CO_2_*) will more likely participate in hydrogenation when H* is available on the adjacent adsorption site (52).

For the hydrogenation process, we find that the active hydrogen atom, H*, detaches from the Fe oxyhydroxide surface and orients toward CO_2_*. This interaction encounters a significant energy barrier, represented by the transition state (TS1), which causes a change in the molecular geometry of CO_2_*, and results in H* connecting to a C atom. As a result, the initial hydrogenation leads to the formation of COOH*, accompanied by a significant increase in free-energy (state 3). Afterward, the Fe oxyhydroxide surface facilitates the activation of H_2_‚ by aiding its dissociation into individual H* which can then participate in subsequent hydrogenation reactions (state 4). Specifically, after overcoming the high energy barrier (TS2), one of these H* coordinates with the COOH* intermediate, forming a C(OH)_2_*, while the other H* is introduced to the α-FeOOH surface to replenish the H* that was previously detached (state 5). Similar hydrogenation reactions with high energy barriers (TS3, TS4) occur when H* coordinates with C(OH)_2_* and HC(OH)_2_* (state 6 and 7), eventually leading to the dehydration and promoting the formation of HCOH* with a highly negative free-energy (state 8). Throughout these transformations, H_2_ acts as a substantial source of H*.

Further, our simulations demonstrate the importance of surface structures of Fe oxyhydroxides and highly reactive HCOH* monomers in promoting C-C chain growth. Fe oxyhydroxides exhibit a compact structure due to the densely packed layers of Fe and O atoms (53). This structure can promote C-C chain growth by providing a high concentration of active sites with more opportunities for the monomers to meet (54, 55). In this study, it is likely that a high concentration of active sites on the Fe oxyhydroxide surface facilitates physical collisions between the HCOH* monomers (state 9). This promotes the formation of the HCOH-HCOH* polymer via C-C chain growth, with a low energy barrier (TS5) and a release of energy in the process (state 10). In condensation reactions that involve CO_2_ conversion to hydrocarbons, the adsorption of CO_2_ on the catalyst surface is a critical step that can determine the selectivity and yield of the reaction (13). The difficulty of CO_2_ adsorption compared to H_2_ on a catalyst surface can lower the C/H ratio at catalytic sites, promoting methane formation instead of longer hydrocarbon chains (56, 57). However, the Fe oxyhydroxide surface shows the adsorption energy of −0.40 eV for CO_2_ (state 1 to state 2), indicating stronger adsorption ability compared to H_2_ (−0.16 eV) (state 3 to state 4), which would thus result in a high C/H ratio (58) on the Fe oxyhydroxide surface favoring organic condensation rather than methane generation.

Fe-based catalysts in the chemical industry are often used to yield linear hydrocarbons (13). The intermediate functional moieties HCOH-HCOH* agree well with our observations which show the presence of alcohol groups within the CCM (Fig. 4). Moreover, CO_2_*, COOH*, and HCOH* are common intermediates for chains to grow (13, 58, 59). Thus, the reactions revealed by DFT simulation provide the potential pathway for initial CO_2_ activation and C-C chain growth, during which the densely packed structure of Fe oxyhydroxide surface and the catalytic redox cycle of H* may have played a key role. Since Fe oxyhydroxides can lose water and transform into hematite, our proposed pathway for C-C chain growth may provide an alternative mechanism for the production of CCM accumulations in the presence of hematite in the Northern Apennine ophiolites (24). Although it remains challenging to identify the organic condensation pathways in hydrothermal systems, our observations, coupled with mechanistic simulations, suggested that surface catalytic mechanisms on Fe oxyhydroxides aid carbonaceous matter formation in altered mafic oceanic crust.

### Implications.

The characterization of abiotic organics is essential for understanding their formation pathways, which are often affected by exogenous contamination. This research is critical for tracing the origin of organic compounds and deciphering a mechanism of CCM formation in the oceanic lithosphere. With the ongoing development of high-resolution molecular geochemical techniques, the discovery of both previously unrecognized and previously known abiotic organic compounds in the oceanic lithosphere, combined with mechanistic simulations, will provide increasingly comprehensive insights into the origin of organic matter and potential formation pathways.

The presence of Fe-catalyzed abiotic organic synthesis in the mafic oceanic crust of the SWIR highlights the importance of recognizing this crust as a potential site for low-temperature organic synthesis, enhancing our understanding of abiotic CCM in the heterogeneous lithosphere. In this process, minerals like Fe oxyhydroxides, which possess densely packed structures and catalytic capabilities, may play a crucial role in organic condensation. Fe oxyhydroxides may have been a primary component in the structures of low-temperature alkaline hydrothermal chimneys, which are posited as potential environments for the origin of life (60, 61). If aliphatic CCM were generated through Fe oxyhydroxides, as hypothesized in this study, they may serve as the fundamental building blocks for more complex biomolecules, such as fatty acids with aliphatic chains, which are essential for life’s emergence. Furthermore, while it is still uncertain whether the abiotic CCM here can serve as potential carbon and nitrogen sources for heterotrophy, our study provides mechanistic insight into the deep carbon cycle.

## Materials and Methods

The crustal rock samples were collected during TS-10 cruise in 2018 (RV Tan Suo Yi Hao) by the human-occupied vehicle (HOV) *Shenhaiyongshi* that was fitted with hydraulically powered manipulators on two swing arms. This specially designed arm for deep-sea conditions can precisely extend and deftly collect samples from the basement. Under the guidance of operators in the HOV, the arm efficiently acquired the samples and stored them safely within a sampling box of the vehicle. Samples were analyzed sequentially to minimize beam-induced damages, in the following order: SEM, Raman, FIB-SEM, PiFM, and TOF-SIMS.

### SEM.

SEM was carried out at Institute of Deep-sea Science and Engineering (IDSSE, Chinese Academy of Sciences), using a Thermo Fisher Scientific Apreo in backscattered electron (BSE) mode. Standard operating conditions for SEM imaging were 2 kV and 0.1 nA, at a working distance of 10 mm. Apreo is equipped with an Edwards XDS10i dry scroll vacuum pump, which prevents oil-based contamination to the SEM chamber.

### Raman Spectroscopy.

Raman spectra were obtained using a WiTec alpha 300R Raman microscope at IDSSE, Chinese Academy of Sciences, using the 532 nm wavelength laser operating at a power less than 0.5 mW with integration times of 10 to 100 s, below the critical dose of radiation that can damage the carbonaceous matter (14, 34). Raman spectra were collected using a 100× objective. Each selected spectrum was fitted using the Fast-Fourier-transform method after subtracting backgrounds using a polynomial function. Assignment of molecular vibrations is based on published data (62, 63).

### FIB-SEM.

FIB-SEM investigations were carried out on Pt-coated specimens in secondary electron and BSE modes under high vacuum, using a FEI Helios 600i at the Analysis and Research Center, Shanghai University, China. Analytical conditions were 2 to 15 kV accelerating voltage, low current (0.1 nA), and a 4 mm working distance. Energy-dispersive X-ray (EDX) spectroscopy analyses were carried out with an Oxford INCA-350 spectrometer. The FIB-SEM was also used to prepare ultrathin foils (final thickness <500 nm). These foils were subsequently analyzed using PiFM and TOF-SIMS.

### PiFM.

The PiFM analysis was performed using a VistaScope microscope (Molecular Vista, Inc.) at IDSSE, Chinese Academy of Sciences. Atomic Force Microscope (AFM) micrographs of varying sizes (0.5 to 50 µm), but set resolution (256 × 256 pixels), were measured in dynamic noncontact mode using NCHAu tips (Nanosensors). Infrared data were collected using a Block Engineering tunable quantum cascade laser (QCL) as mid-IR source providing an IR working range of 775 to 1,970 cm^−1^.

After acquiring topography information, IR information was collected in two forms: 1) single-point IR spectra, 2) IR images at set wavenumbers. The single-point IR spectra were taken with an acquisition time of 60 to 200 s, a resolution of 4 cm^−1^. These near-field infrared measurements were performed with an approximate lateral resolution of 10 nm and a probing depth of 30 nm (64). The IR images were taken after identifying relevant IR wavenumbers from the point spectra. IR images were taken simultaneously with topography images by tuning detection of the topography signal to the second-, and IR signal to the first Eigenmode of the AFM cantilever. The PiFM data (AFM micrographs, point spectra, IR images) was then processed using the SurfaceWorks software (Molecular Vista, Inc.).

### TOF-SIMS.

TOF-SIMS experiments were conducted using a TOF-SIMS V reflection-type mass spectrometer (IONTOF GmbH) at Suzhou Institute of Nano-Tech and Nano-Bionics (SINANO, Chinese Academy of Sciences). The instrument is equipped with a liquid metal ion gun (LMIG) and a dual-source ion column (DSC-S). The LMIG source was used as the primary ion beam to deliver a pulsed Bi_3_^+^ cluster at 30 keV. The DCS-S source, providing Cs^+^ and O_2_^+^, was used for ultra-low energy sputtering. The primary ion beam was at a repeating frequency of 10 kHz, covering the secondary ion mass range of 0 to 916 Da in both positive and negative spectra. To remove surface contaminations before measurements, surface cleaning with the Cs^+^ and O_2_^+^ sputter beam was performed at 500 eV for negative and positive modes, respectively. Spectra of a larger area of 50 µm × 50 µm were acquired in the spectrometry mode (Bunched mode) with a pulse current of 0.7 pA. In bunched mode, the mass-resolution (m/Δm) of the secondary ion peak was between 6,000 and 9,000. Subsequently, a field of interest with a size of 20 µm × 20 µm was further scanned to obtain intensive images in fast imaging mode (Burst alignment mode) with a pulsed current of 0.01 pA, resulting in a spatial resolution of 200 nm. Ion images were acquired with 128 × 128 pixel^2^ in both negative and positive polarities of the feature field, giving a pixel size of 156 × 156 nm^2^. In spectrometry mode, total scans of 100 and 200 were acquired separately in positive and negative polarities. In fast imaging mode, 1,800 and 1,000 scans were adopted in positive and negative polarities, respectively. The images were recorded with a primary ion fluence of 2.56 × 10^12^ ions/cm^2^ (100 scans with a cycle time of 100 μs), which was kept below the static SIMS limit. Typically, TOF-SIMS is extremely sensitive and can achieve detection limits at the ppm (parts per million) or ppb (parts per billion) level (less than 1 ppm) for organic molecules (65). Data processing was done using the SurfaceLab 6.7 software (IONTOF GmbH) at Macau Institute for Applied Research in Medicine and Health, Macau University of Science and Technology, China. The mass calibration was carried out with the ions of C^+^, Na^+^, Cu^+^, Cu_2_^+^, Cu_3_^+^, Cu_4_^+^, Cu_5_^+^ in positive spectra and the ions of CH^−^, CH_2_^−^, C_4_^−^, Cu^−^, Cu_2_^−^, Cu_3_^−^, Cu_4_^−^ in negative spectra of the interested area. These ions were chosen for their well-characterized mass-to-charge ratios to ensure accurate mass determination across the spectra. Moreover, the spectra of the feature area were internally calibrated using ion of C^+^, Na^+^, Fe^+^, Cu^+^ in positive polarity and the ion of C^−^, CN^−^, S^−^, CuO^−^, Pt^−^ in negative polarity. Spectra from the total analysis area or selected regions of interest were further extracted. Assignments of ion peaks were performed according to the instrument resolution, accuracy, and the valence rule.

### Quantum Mechanical Modeling.

First-principles calculations were carried out under the scheme of spin-polarized DFT using CASTEP (66). Specifically, the Perdew–Burke–Ernzerhof (PBE) exchange-correlation functional (67) within the generalized gradient approximation was employed to describe the exchange-correlation energy. A goethite (001) surface model was employed, with cell parameters: *a* = 9.95 Å, *b* = 3.02 Å, *c* = 4.60 Å, *α* = *β* = *γ* = 90°; space group: Pnma. Geometric convergence tolerances were set for a maximum force of 0.03 eV/Å, a maximum energy change of 10 to 5 eV/atom, a maximum displacement of 0.001 Å and maximum stress of 0.5 GPa. In addition, the sampling within the Brillouin zone was set to 3 × 3 × 1 by the Monkhorst-Pack method. Hydrogen capture by CO_2_ was investigated by searching the possible hydrogen transfer route and identifying the migration transition state with the lowest diffusion energy barrier. The energy barrier is the energy difference between the total energies of the transition state and the initial structure. The transition state is searched by the generalized synchronous transit (LST/QST) method (68) implemented in the CASTEP code. The algorithm starts from a linear synchronous transit (LST) optimization and continues with a quadratic synchronous transit (QST) maximization process. After that, the conjugate gradient (CG) minimization is conducted from the obtained LST/QST structure to refine the geometry of the transition state. The LST/QST/CG calculations are repeated until a stable transition state is obtained.

## Supplementary Material

Appendix 01 (PDF)

Dataset S01 (XLSX)

Dataset S02 (XLSX)

## Data Availability

All study data are included in the article and/or supporting information.
