# Supplementary material for: Unraveling abiotic organic synthesis pathways in the mafic crust of mid-ocean ridges
Source: Proc Natl Acad Sci U S A. 2024 Oct 10;121(43):e2308684121. doi: 10.1073/pnas.2308684121 (PMC11513914; doi:10.1073/pnas.2308684121)
Supplement: Supplementary file 1 — Appendix 01 (PDF) [file pnas.2308684121.sapp.pdf]

Supporting Information for

Unraveling abiotic organic synthesis pathways in the mafic crust of mid-ocean ridges

Jingbo Nan (南景博)[a,b,c,1], Xiaotong Peng (彭晓彤)[a,2], Oliver Plümper[d], Iris C. ten Have[e], Jing-Guang Lu[f], Qian-Bao Liu[f], Shao-Lin Li[g], Yingjie Hu[h], Yu Liu[i], Zhen Shen[i], Weiqi Yao[j], Renbiao Tao[c], Martina Preiner[k,l,m,d], Yongxiang Luo[a]

[a]Institute of Deep-sea Science and Engineering, Chinese Academy of Sciences, 572000 Sanya, China
[b]Nanjing Institute of Geology and Palaeontology, Chinese Academy of Sciences, 210008 Nanjing, China
[c]Center for High Pressure Science and Technology Advanced Research, 100094 Beijing, China
[d]Department of Earth Sciences, Faculty of Geosciences, Utrecht University, 3584 CD Utrecht, the Netherlands
[e]Debye Institute for Nanomaterials Science, Faculty of Science, Utrecht University, 3584 CG Utrecht, the Netherlands
[f]State Key Laboratory of Quality Research in Chinese Medicines, Macau Institute for Applied Research in Medicine and Health, Macau University of Science and Technology, Taipa, Macau, China
[g]The State Key Laboratory of Lunar and Planetary Science, Macau University of Science and Technology, Taipa, Macau, China.
[h]Nanjing Key Laboratory of Advanced Functional Materials, Nanjing Xiaozhuang University, 211171 Nanjing, China
[i]College of Geoscience and Surveying Engineering, China University of Mining and Technology (Beijing), 100083 Beijing, China
[j]Department of Ocean Science and Engineering, Southern University of Science and Technology, 518055 Shenzhen, China
[k]Microcosm Earth Center, Max Planck Institute for Terrestrial Microbiology and Philipps Universität Marburg, Germany.
[l]Center for Synthetic Microbiology (SYNMIKRO), Marburg, Germany.
[m]Geochemical Protoenzymes Research Group, Max Planck Institute for Terrestrial Microbiology, Germany.
[1]Present address: Nanjing Institute of Geology and Palaeontology, Chinese Academy of Sciences, 210008 Nanjing, China
[2]To whom correspondence may be addressed. Email: xtpeng@idsse.ac.cn

This PDF file includes:

Supporting Information Text
Figures S1 to S11
Table S1 to S3
SI Methods
SI References

Other supporting materials for this manuscript include the following:

Datasets S1
Datasets S2

Supporting Information Text

Geological setting and samples selection

The SWIR is an ultraslow-spreading mid-ocean ridge (~14 mm/yr) located along the floor of the Indian Ocean (1). It separates the Somali and Antarctic plates and presents a V-shaped outline, resulting from the interaction between the ridge and surrounding hot spots (2). Deep fracture zones characterise the SWIR. These fracture zones have continuous subparallel trends and extend to a depth of 2,000 metres below sea-level (mbsl) (3). Continuous tectonic processes along the SWIR have led to serpentinite- and basalt-hosted hydrothermal systems (4). Among them, the Dragon Horn area and Tian Cheng area are located on the south flank of the SWIR (Fig. S1), consisting of several mafic and ultramafic rock-hosted hydrothermal vents (5). The samples studied here were recovered from the two areas by the human-occupied vehicle (HOV) *Shenhaiyongshi* during Dive 122 and Dive 089 (TS-10 cruise in 2018, RV Tan Suo Yi Hao) at 2633-2811 meters below sea level (mbsl).

The presence of Fe oxyhydroxides without hydrous phyllosilicates

The upper oceanic crust predominantly consists of basaltic lava erupted and solidified at mid-ocean ridges. Hydrothermal alteration due to fluid circulation typically leads to the release of Fe and alkali elements, forming common secondary minerals such as Fe oxyhydroxides and hydrous phyllosilicates, often observed in highly altered basalts. This process is especially prevalent on the older ridge flanks, where seawater circulation in the upper oceanic crust results in the infilling of fractures and veins with hydrous phyllosilicates, significantly reducing porosity and permeability (6). However, our studied basalts are relatively fresh (Fig. S2), with only localized alteration. In this context, the concentrations of reactive elements (e.g., Fe, alkali elements) released during alteration would be relatively low, making it difficult to form secondary minerals. Moreover, compared to Fe, alkali elements are more mobile and easily leached from basalts (7), making the formation of hydrous phyllosilicates even more challenging compared to Fe oxyhydroxides. Experimental studies on olivine weathering simulating seafloor conditions have shown that Fe oxyhydroxides tend to form before secondary phyllosilicates (e.g., sepiolite, talc) because ferrous iron released from olivine is rapidly oxidized to ferric iron, which then precipitates (8). Thus, the rapid precipitation of Fe oxyhydroxides, combined with the high mobility and leaching of alkali elements, impedes the formation of hydrous phyllosilicates. While Fe oxyhydroxides can result from aqueous alteration, the conditions and extent of alteration in our samples are insufficient to support the concurrent formation of hydrous secondary silicates.

Alternatively, the ascent of Fe-rich but alkali-poor fluids from the deep lithosphere to shallower crustal levels could explain the presence of Fe oxyhydroxides without hydrous phyllosilicates in the studied basalts. Previous study reported that the dissolved Fe concentration in the Dragon Horn area hydrothermal fluids is twice as high as that in the Trans-Atlantic Geotraverse (TAG) hydrothermal fields on another slow-spreading ridge (5). This elevation in dissolved Fe levels results from extensive water-rock interactions within the deep lithosphere, facilitated by deep fault systems prevalent in this area and enhanced by high chloride concentrations that increase Fe solubility. In contrast, concentrations of alkali elements remain at the same level (5). As these Fe-rich fluids ascend to shallower crustal levels and cool, they may encounter oxidizing conditions near the subseafloor, leading to the precipitation of Fe oxyhydroxides within basaltic rocks. This phenomenon is also commonly observed around hydrothermal vents (9, 10).

Structural variations among goethite (α-FeOOH), akaganeite (β-FeOOH), and lepidocrocite (γ-FeOOH)

Goethite has an orthorhombic unit cell where each Fe^{3+} ion is octahedrally coordinated by three O^{2-} ions and three OH^- ions, forming a slightly distorted octahedral structure (11). This mineral has double chains of edge-sharing FeO_6 octahedra running parallel to one another. Akaganeite is tetragonal, characterized by its structure of edge-shared FeO_6 octahedra that form tunnel-like structures. These tunnels can accommodate chloride ions and water molecules. Unlike goethite and akaganeite, which are characterized by their octahedral chains and tunnels, lepidocrocite is a layered compound. Its structure consists of sheets formed by arrays of O^{2-} and OH^- anions, with

Fe^{3+} ions occupying the octahedral interstices between these sheets. The presence of Fe in various oxidation states and coordination environments (e.g., edge-sharing octahedra in goethite) provides versatile and accessible active sites for catalysis.

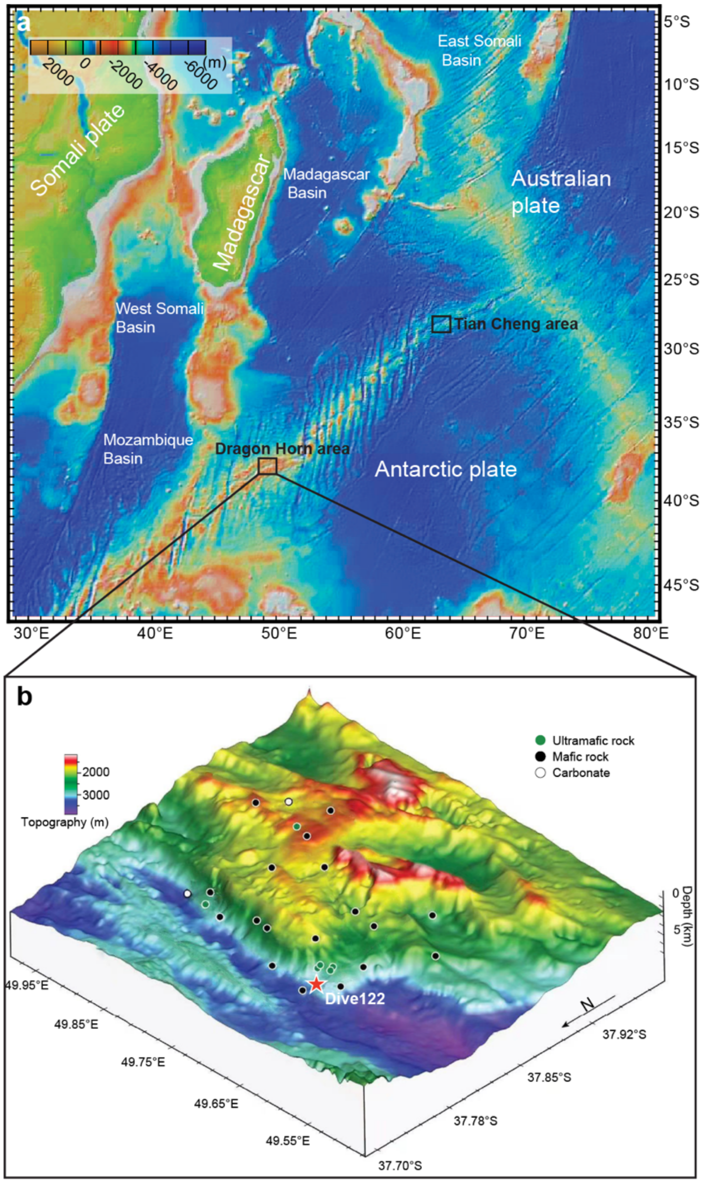

Fig. S1. Location of the Dragon Horn area and Tian Cheng area at the Southwest Indian Ridge. a Bathymetry map of the Southwest Indian Ridge (SWIR) and sampling site. The black box depicts the sampling location in Dragon Horn area (37°46.8′S, 49°38.8′E, 2780 mbsl) executed by HOV *Shenhaiyongshi*. The background map was made using GeoMapApp (http://www.geomapapp.org/). b Three-dimensional view of the Dragon Horn area (modified from ref. (5), depicting the location of sampling site during Dive 122 shown as the red star.

Table S1 Information of the crustal rocks recovered from the SWIR. The proportions of pyroxene, plagioclase, and olivine are calculated using XRD (Datasets S2), while Fe oxyhydroxide, Fe-Ti oxide, and sulphide are estimated through observation. Volume fraction of fractures and vesicles is calculated from CT results (also see Fig. S7)

Hydro-thermal system	Sample ID	Image	Depth (mbsl)	Longitude & latitude	CCM detected	Mineralogy	Volume fraction of fractures &vesicles
Dragon Horn	SY122-G06	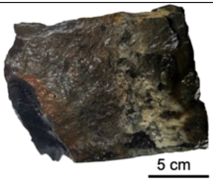	2780	49.647°E<br>37.780°S	Yes	Pyroxene (52 vol.%), plagioclase (27 vol.%), olivine (18 vol.%), Fe oxyhydroxide (2 vol.%), Fe–Ti oxide and sulfide (<1 vol.%)	3.21 vol.%
Tian Cheng	SY089-G02	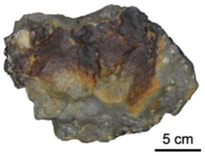	2811	63.832°E<br>27.851°S	Yes	Plagioclase (68 vol.%), pyroxene (23 vol.%), olivine (6 vol.%), Fe oxyhydroxide (2 vol.%), Fe–Ti oxide and sulfide (<1 vol.%)	2.56 vol.%
Tian Cheng	SY089-G03	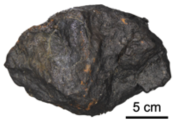	2750	63.729°E<br>27.291°S	No	Plagioclase (68 vol.%), Pyroxene (20 vol.%), olivine (9 vol.%), Fe oxyhydroxide (1 vol.%), Fe–Ti oxide and sulfide (<1 vol.%)	1.65 vol.%
Tian Cheng	SY089-G04	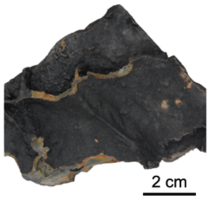	2633	63.743°E<br>27.205°S	No	Plagioclase (67 vol.%), pyroxene (24 vol.%), olivine (6 vol.%), Fe oxyhydroxide (2 vol.%), Fe–Ti oxide and sulfide (<1 vol.%)	2.39 vol.%
Tian Cheng	SY089-G08	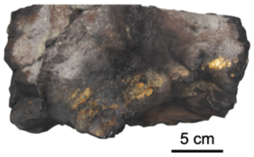	2742	63.962°E<br>27.852°S	No	Plagioclase (66 vol.%), pyroxene (22 vol.%), olivine (10 vol.%), Fe oxyhydroxide (1 vol.%), Fe–Ti oxide and sulfide (<1 vol.%)	1.06 vol.%
Dragon Horn	SY120-G04	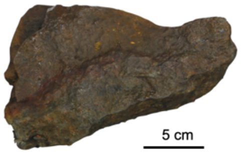	2761	50.466°E,<br>37.657°S	No	Pyroxene (74 vol.%), olivine (24 vol.%), Fe oxyhydroxide (<1 vol.%), Fe–Ti oxide and sulfide (<1 vol.%)	0.38 vol.%
Dragon Horn	SY120-G05	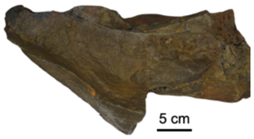	2766	50.467°E<br>37.658°S	No	Plagioclase (51 vol.%), pyroxene (35 vol.%), olivine (12 vol.%), Fe oxyhydroxide (1 vol.%), Fe–Ti oxide and sulfide (<1 vol.%)	2.24 vol.%

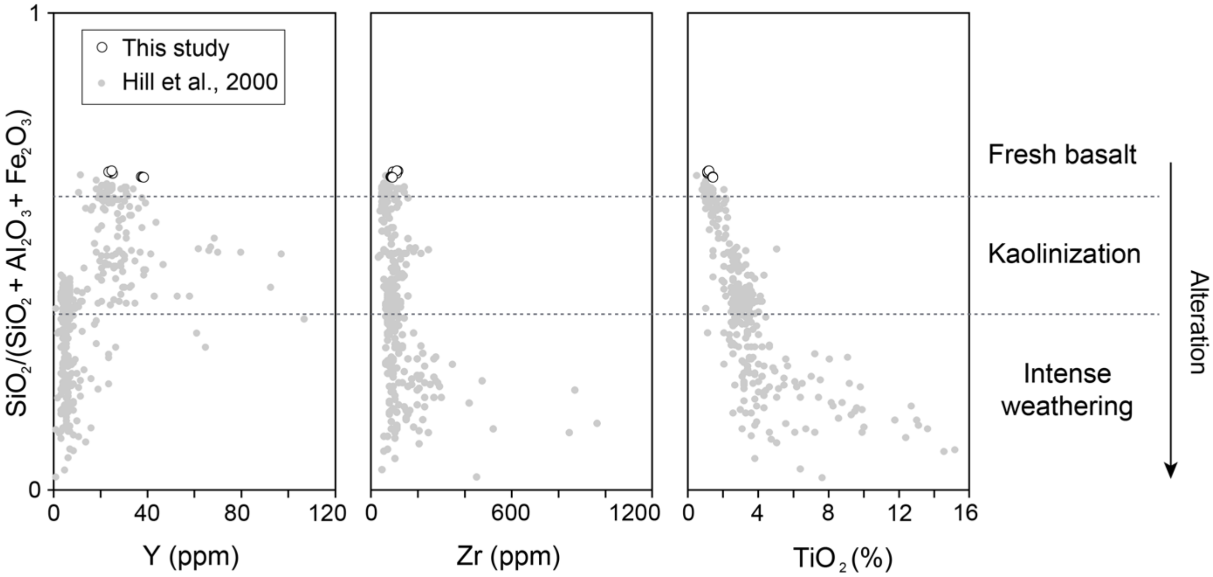

Fig. S2 Variation diagrams for major and trace elements of this study and Hill et al. (2000). Yttrium (Y), zirconium (Zr), and TiO_2 are plotted against the ratio of SiO_2 to (SiO_2 + Al_2O_3 + Fe_2O_3) to demonstrate that while TiO_2 exhibits a relative increase, yttrium shows a marked decrease when alteration increases. The studied basalts are located within the fresh zone, prior to the onset of kaolinization. Please also see Table S2.

Table S2. Elemental data for the studied basalts

Compound	SY122-G06	SY089-G02	SY089-G03	SY089-G04	SY089-G08	SY120-G04	SY120-G05
SiO_2 (wt%)	49.33	50.43	50.92	50.59	49.92	49.32	49.56
TiO_2 (wt%)	1.42	1.13	1.14	1.16	1.19	1.40	1.40
Al_2O_3 (wt%)	14.34	18.14	18.15	17.75	17.09	14.71	14.85
Fe_2O_3 (wt%)	11.47	7.26	7.56	7.69	7.94	11.45	11.41
Y (ppm)	38.20	24.40	25.00	23.80	24.60	37.20	37.80
Zr (ppm)	95.00	104.00	102.00	97.70	102.00	92.30	93.60

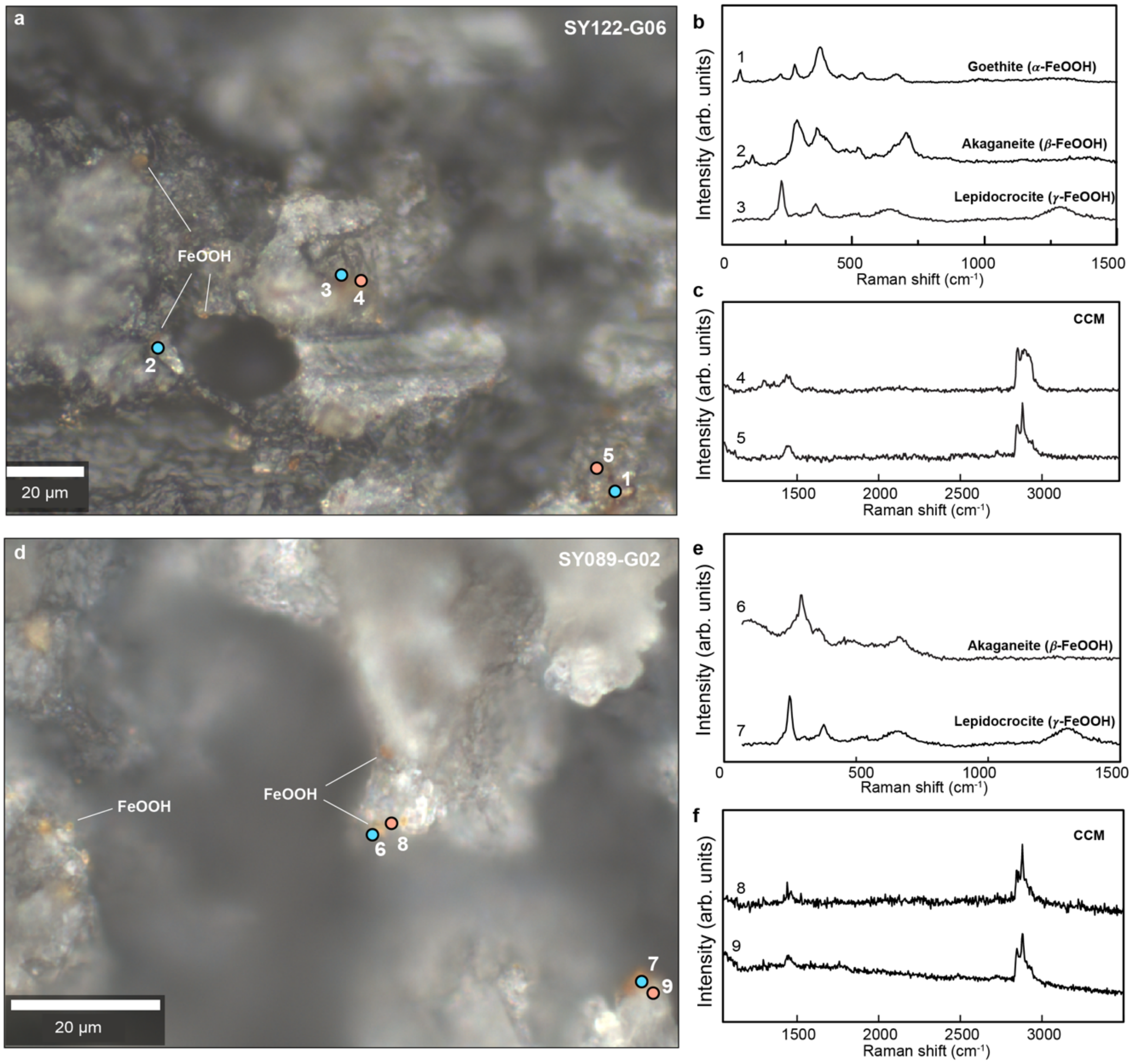

Fig. S3. The presence of scattered Fe oxyhydroxides (FeOOH) in SY122-G06 and SY089-G02 basalt samples. a. and d. Reflected light photomicrography of representative unpolished rough surface of the basalts, showing the presence of reddish Fe oxyhydroxides. b. and e. Raman spectra for three kinds of Fe oxyhydroxides in the studied basalts. Assignments are based on ref (14, 15). c. and f. Raman spectra for the condensed carbonaceous matter (CCM) associated with Fe oxyhydroxides.

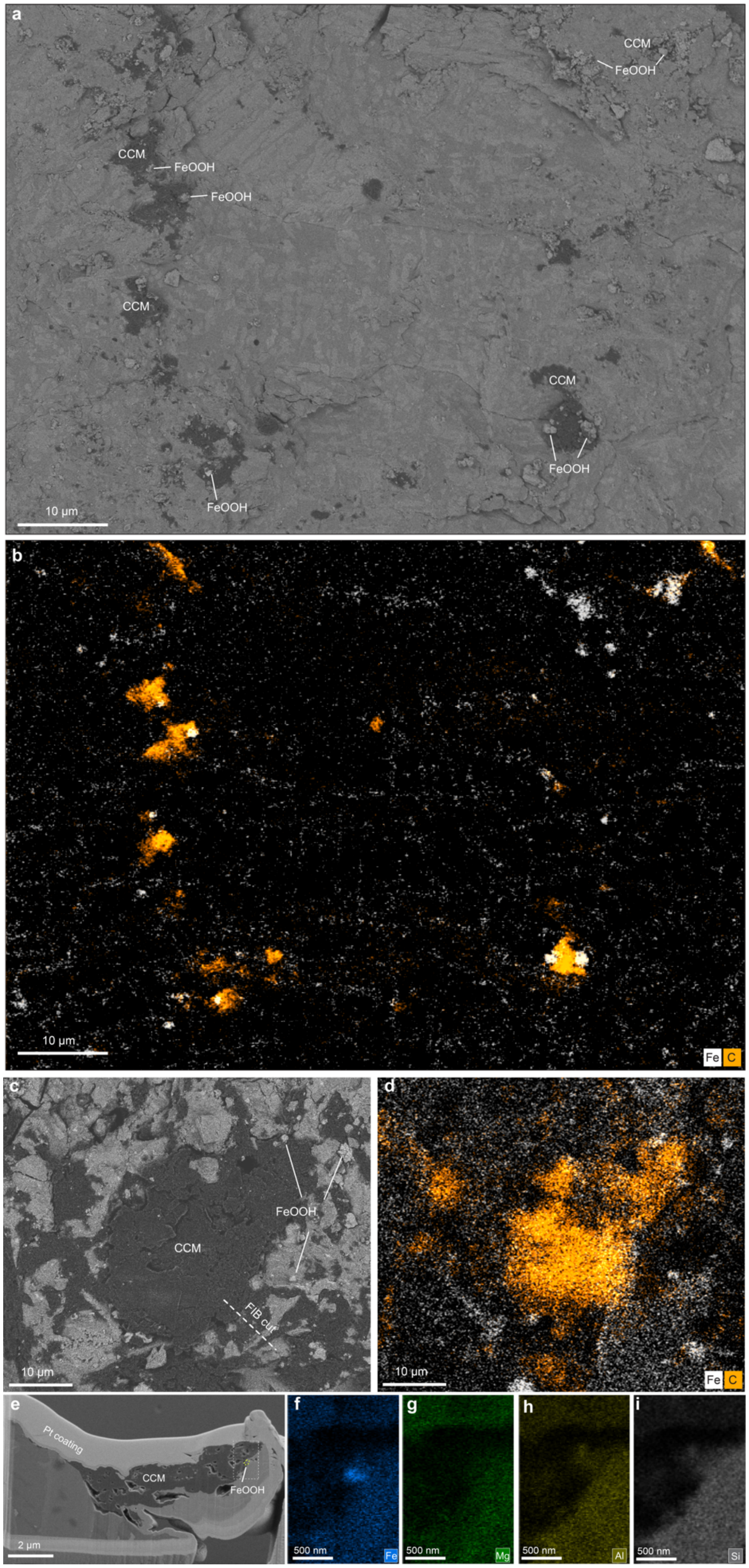

Fig. S4. Association of condensed carbonaceous matter (CCM) with Fe oxyhydroxide (FeOOH) in SY122-G06 sample.

a. and c. Back-scattered electron image of CCM patches, and their related Fe oxyhydroxides. Dashed line shows focused ion beam foil location in Fig 2a in the main text.

b. and d. Corresponding energy-dispersive X-ray spectrometry map of a. and c., respectively, showing the elemental distributions of carbon (orange) and iron (white).

e. SEM image of the FIB foil displayed in c and Fig 2a, and its corresponding elemental mapping in f-i. Light blue color in f. indicates the presence of Fe oxyhydroxide (FeOOH).

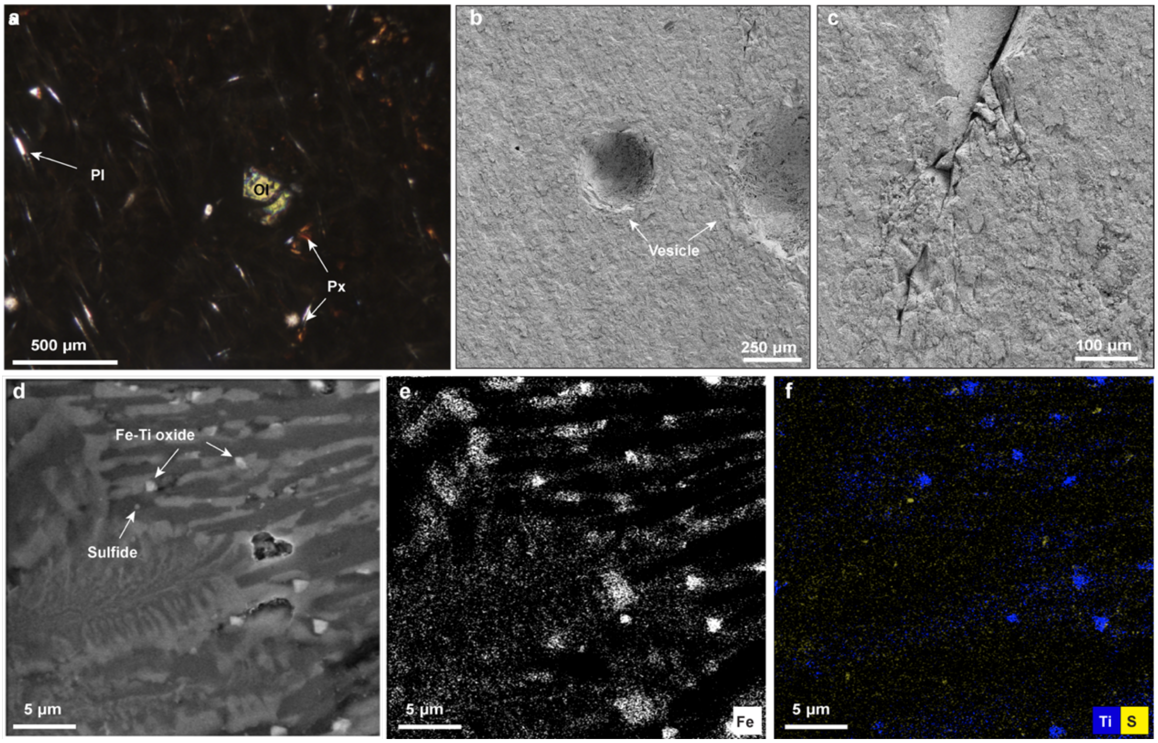

Fig. S5. Micro-optical and SEM images of the basalt sample SY122-G06. a. Cross-polarized transmitted light photomicrograph of thin section from basalt which is composed of phenocrysts of pyroxene, olivine and plagioclase. b-c. Secondary electron image showing the vesicles in b and fracture network in c. d. Back-scattered electron image of matrix with scattered sulfides and Fe-Ti oxides. Pl, plagioclase; Px, pyroxene; Ol, olivine. e-f. Corresponding energy-dispersive X-ray spectrometry map of c, showing the elemental distributions of iron (white), sulfur (yellow) and titanium (blue).

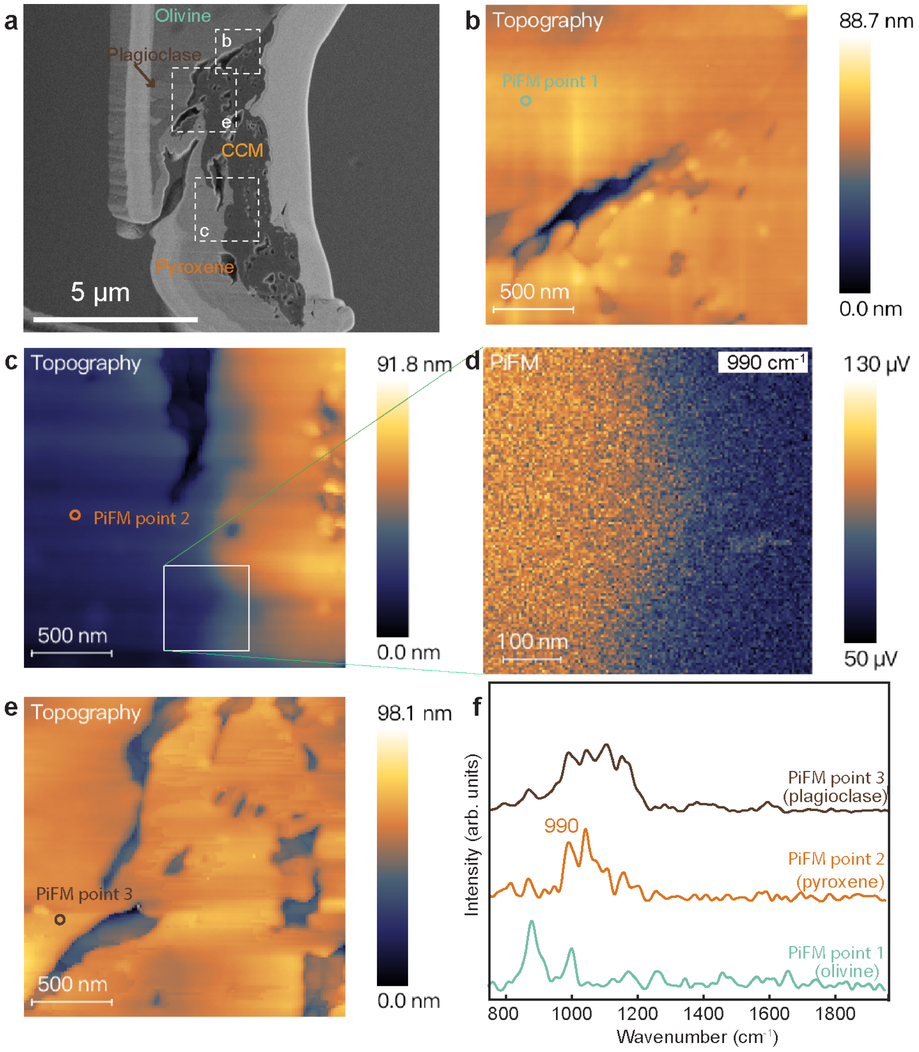

Fig. S6. PiFM analysis of mineral assemblage within basaltic matrix from sample SY122-G06. a. Rotated SEM image of the FIB foil displayed in Fig. 2a. b, c, e. Topographic images of FIB foil shown in a. d. IR map corresponding to large view in c at 990 cm^{-1} (indicative for pyroxene). f. PiFM-IR spectra collected from b-e, assignments are based on ref (12, 13).

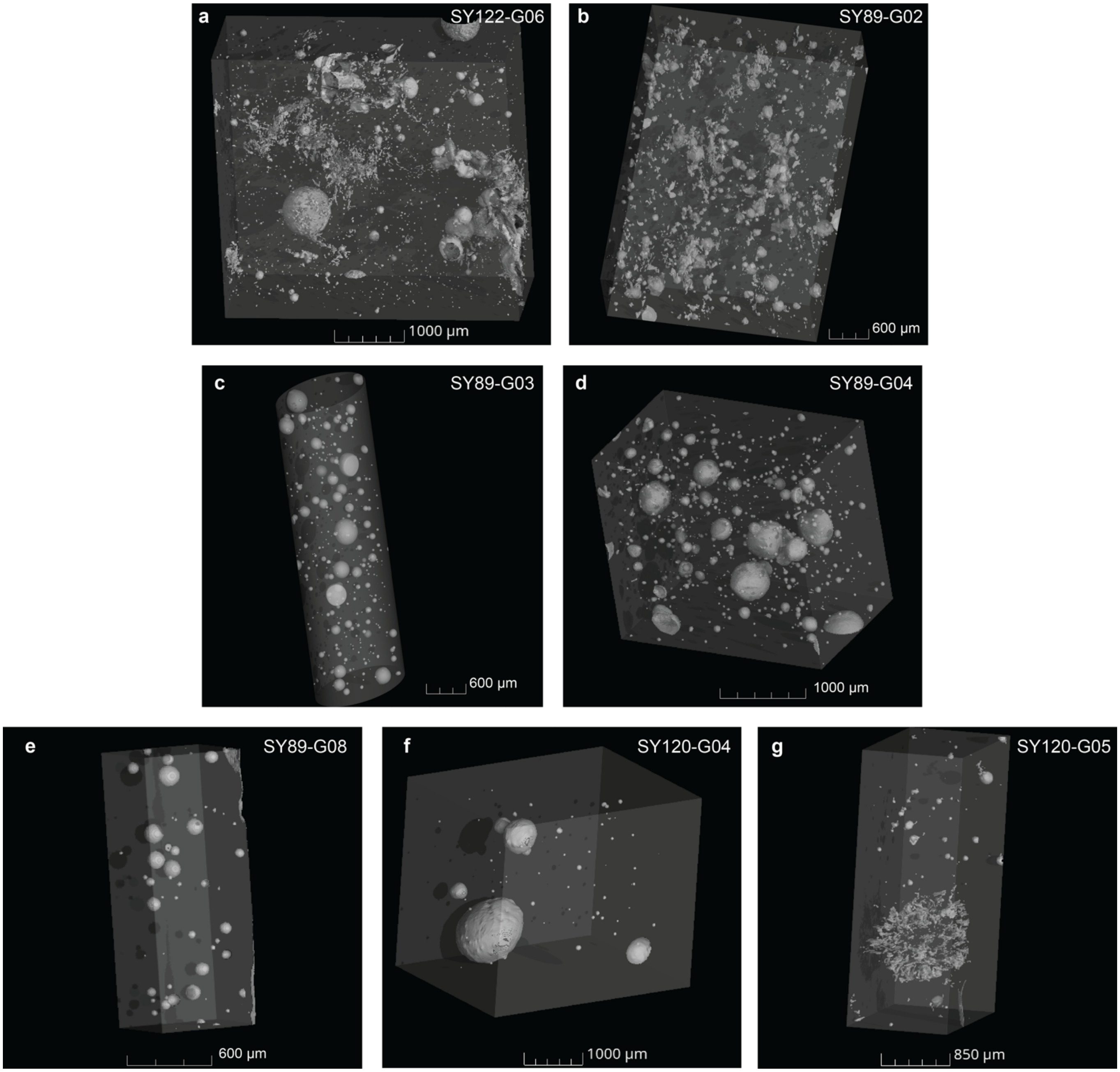

Fig. S7 The X-ray computed tomography scan of the basalt samples provided complete reconstructed 3D images, with light grey areas representing fractures and vesicles**.**

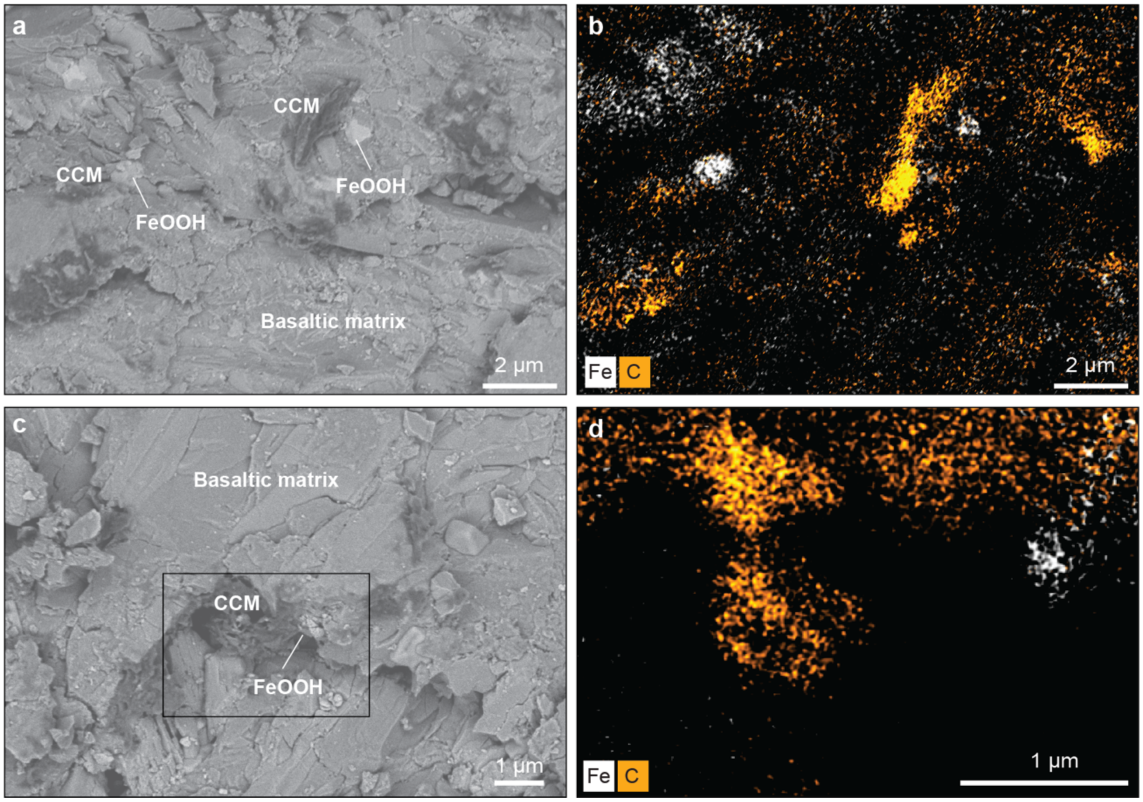

Fig. S8 Association of condensed carbonaceous matter (CCM) with Fe oxyhydroxide (FeOOH) in SY089-G02 sample. a. and c. Back-scattered electron image of CCM patches in the basaltic matrix. b. and d. Corresponding energy-dispersive X-ray spectrometry map of a. and c. showing the elemental distributions of carbon (orange) and iron (white).

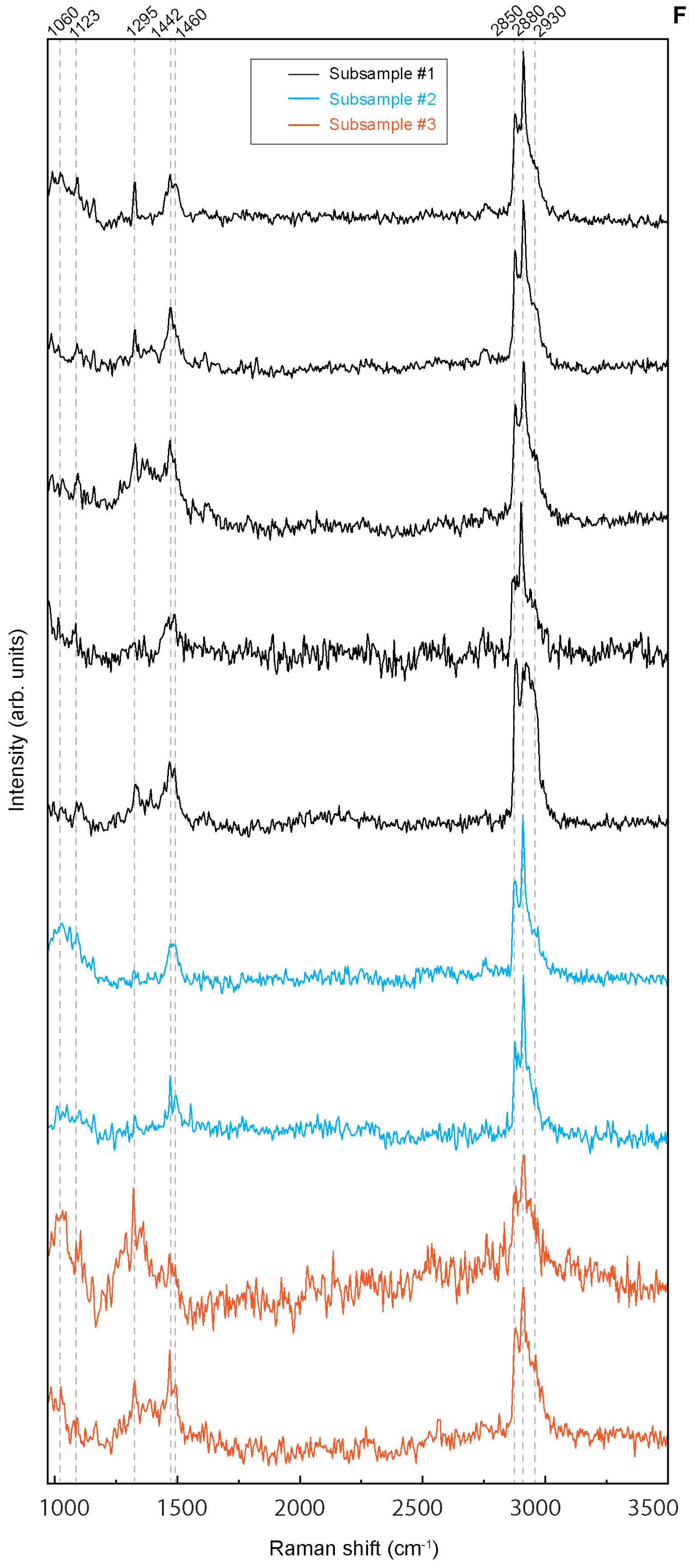

Fig. S9. Randomly collected Raman spectra for CCM basalt subsamples from SY122-G06. Each of the spectra is from a different CCM occurrence. Subsample #1 was cut without polishing, while Subsample #2 and #3 were freshly opened without cutting, showing the pristine surface. These slightly diversified spectra corroborate the homogeneous distribution of aliphatic compounds.

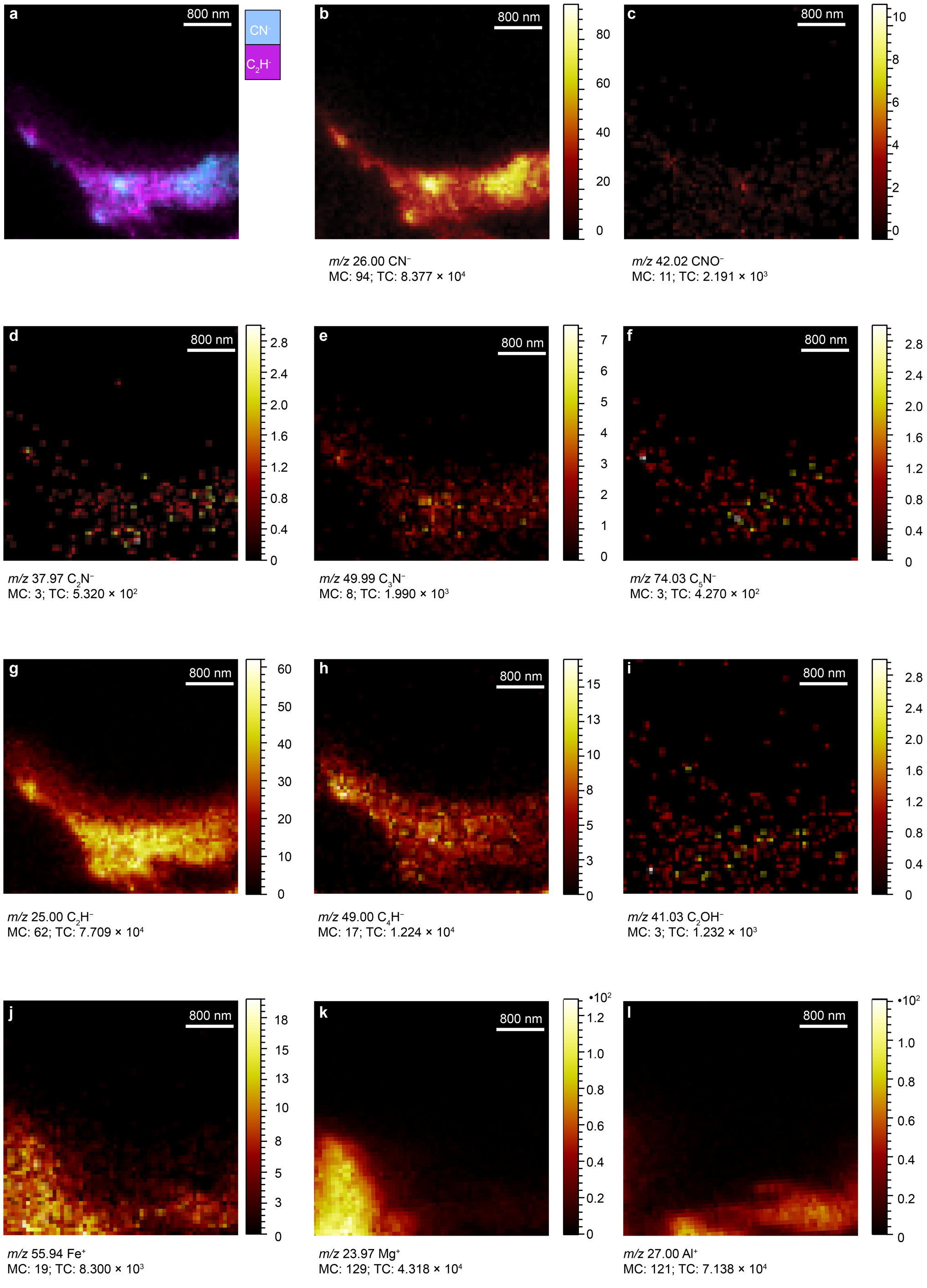

Fig. S10. TOF-SIMS ion images of FIB foil from sample SY122-G06, showing the presence of aliphatic compounds and related minerals. a. Association of C_2H^- (purple) and CN^- (blue) displayed in Fig 3. b-f. The distribution of amines and alcohols, charactered by CN^-, CNO^-, C_2N^-, C_3N^-, C_5N^- separately. g-i. The distribution of alkanes and alcohols, charactered by C_2H^- (at 26.0 m/z), C_4H^-, C_2OH^- separately. j-l. Ion image of Fe^+, Mg^+ and Al^+, showing the location of basaltic matrix displayed in Fig. 2a.

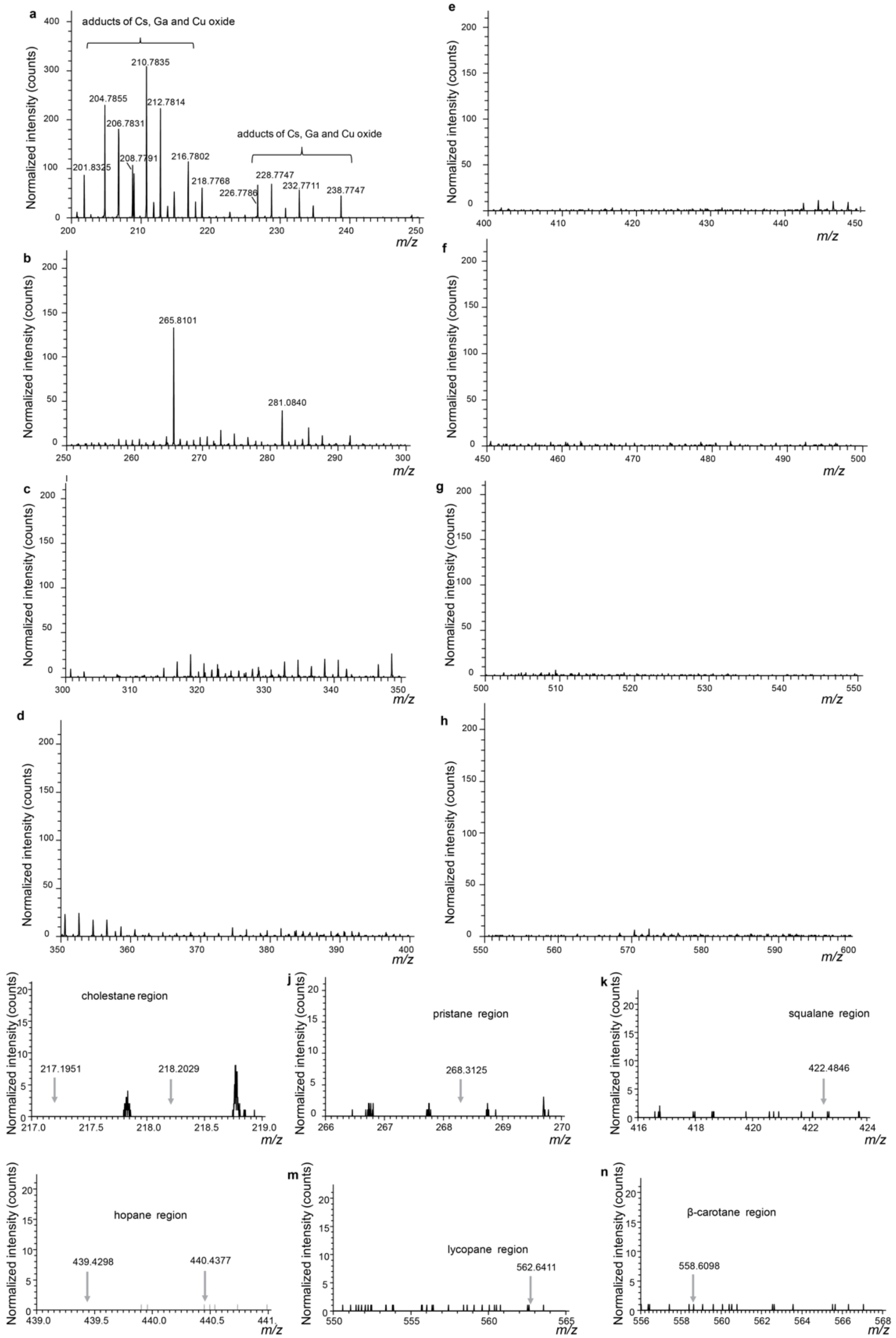

Fig. S11. TOF-SIMS spectra collected in the CCM showing the absence of common biomarkers. a-h, enlarged views of the TOF-SIMS spectrum displayed in Fig. 2b. i-n, selected magnified view of this TOF-SIMS spectrum showing regions in which the peaks of fragment ions characteristic of common biomarkers such as pristane ($C_{19}H_{40}$), cholestane ($C_{27}H_{48}$), squalene ($C_{30}H_{50}$), lycopane ($C_{40}H_{56}$), β-carotene ($C_{40}H_{56}$) and hopanoids should lie. However, these common biomarkers are absent in the TOF-SIMS spectrum. Please see ref (16–19) for details.

Table S2. Assignment for the PiFM-IR bands of CCM within basaltic matrix shown in Fig. 3b. Assignments are based on ref (20, 21).

No.	Wavenumber (cm^{-1})	Assignment
1	880	C-H bending
2	1420	alcohol O-H bending
3	1462/1472	CH_2/CH_3 stretching
4	1085	alcohol C-O stretching (primary)
5	1115	alcohol C-O stretching (secondary)
6	1230	amine C-N stretching
7	1344	amine C-N stretching (conjugated)
8	1422	alcohol O-H bending
9	1580-1650	amine N-H bending

SI Methods

X-ray diffraction

The XRD measurements were performed at Guangzhou Institute of Geochemistry, Chinese Academy of Sciences, on a BRUKER D8 ADVANCE X-ray diffractometer, with a Cu target (λ=0.15406 nm) and a Ni filter. The instrument operated at a tube voltage of 40 kV and a tube current of 100 mA, with continuous scanning mode. Scanning range was from 3° to 70° (2θ) at a scanning speed of 8°/min. Samples were powdered to <70 μm using an agate crusher, and then were homogenized, sieved and sub-sampled for XRD analyses. Peak identification was conducted with automatic baseline determination to minimize subjective influences. Representative diffraction peaks were selected for area measurement. The volume proportion of mineral phases was calculated by measuring the area under these peaks and using the K value to account for variations in orientation among different minerals. This semiquantitative estimate for each identified mineral is based on its relative K value, providing a measure of the relative amounts of different major mineral phases (>5%) within the studied basalts.

Element analysis

Major element analyses were analyzed using the Rigaku ZSX Primus II X-ray Fluorescence Spectrometer, which is operated at a power of 4KW. The sample preparation involved the accurate weighing of 4g of rock powder, previously dried at 105°C, to a precision of 0.01g. This powder was transferred into a pellet mold and boric acid was added to edge the sample. A press machine capable of exerting pressures greater than 25 MPa was used to apply a force of 30 MPa for 3 seconds to form the pellets. The surfaces of the resulting pellets were cleaned of any residual powder using a bulb syringe, and the pellets were labeled for identification during analysis. To ensure the accuracy and reliability of the results, standard reference materials such as GSD-9, GSD-11, and GSD-12 were employed in the quality control protocol. The precision of the measurements was maintained within a 2% deviation, confirming the consistency of the data. Throughout the experimental process, duplicate samples were prepared every five tests to monitor and maintain the stability of the analytical procedure.

Trace element analyses were performed using a Thermo Fisher iCAPQ ICP-MS equipped with a ASX560 auto-sampler. Approximately 50 mg of each sample powder (<70 μm) was dissolved in Teflon bomb with the double-distilled concentrated HNO_3-HF (1:1) mixture. The dissolution was maintained in an oven at 185°C for 3 days and then dried down to evaporate HF. The residues were re-dissolved with double-distilled concentrated HNO_3 followed by the HNO_3-H_2O (1:1) mixture, and dried again. After that, the sample was dissolved in the final 3 mL 2N HNO_3 stock solution. Finally, the sample solution was diluted by 1000 times with 2.5% HNO_3 and 10ppb Rh internal spikes. Multiple element calibration standards were used. Internal spikes and external monitors were used to correct instrument drift mass bias.

X-ray computed tomography

The CT analysis was conducted at the Micro-CT lab (NIGPAS) using a Zeiss Xradia 520 Versa system. The CT scans were performed with an operating voltage of 60 kV for the X-ray tube and utilized a thicker LE3 filter to minimize beam-hardening artifacts. This setup facilitated non-destructive 3D reconstruction, allowing for detailed visualization of the structures of vesicles and fractures within the basalts. Each scan comprised 801 equiangular projections captured over a full 360° rotation, with an exposure time of 2 seconds per projection. The volumetric data obtained were subsequently analyzed and processed using VG Studio Max 4.0 software.

SI References

1. C. DeMets, R. G. Gordon, D. F. Argus, S. Stein, Current plate motions. *Geophys. J. Int.* **101**, 425-478 (1990).
2. D. Sauter, et al., Propagation of a melting anomaly along the ultraslow Southwest Indian Ridge between 46°E and 52°20′E: Interaction with the Crozet hotspot? *Geophys. J. Int.* **179**, 687-699 (2009).
3. J. Y. Royer, P. Patriat, H. W. Bergh, C. R. Scotese, Evolution of the Southwest Indian Ridge from the Late Cretaceous (anomaly 34) to the Middle Eocene (anomaly 20). *Tectonophysics* **155**, 235-260 (1988).
4. W. Bach, N. R. Banerjee, H. J. B. Dick, E. T. Baker, Discovery of ancient and active hydrothermal systems along the ultra-slow spreading Southwest Indian Ridge 10°-16°E. *Geochem. Geophy. Geosys.* **3**, 1-14 (2002).
5. C. Tao, et al., Deep high-temperature hydrothermal circulation in a detachment faulting system on the ultra-slow spreading ridge. *Nat. Commun.* **11**, 1300 (2020).
6. W. Bach, and G. L. Fruh-Green, Alteration of the oceanic lithosphere and implications for seafloor processes. *Elements*, **6**, 173-178 (2010).
7. A. Aiuppa et al., Mobility and fluxes of major, minor and trace metals during basalt weathering and groundwater transport at Mt. Etna volcano (Sicily). *Geochim. Cosmochim. Acta.* **64**, 1827-1841 (2000).
8. M. Fuhr et al., Kinetics of olivine weathering in seawater: an experimental study. *Frontiers in Climate*. **4**, 831587 (2022).
9. K. Ta, Z. Wu, X. Peng, Z. Luan, and S. Chen, Formation and origin of Fe–Si oxyhydroxide deposits at the ultra-slow spreading southwest indian ridge. *Deep-Sea Res. I: Oceanogr. Res. Pap*, **170**, 103491 (2021)
10. C. Gini et al., Iron oxyhydroxide-rich hydrothermal deposits at the high-temperature Fåvne vent field, Mohns Ridge. *Geochem. Geophy. Geosys.* **25**, e2024GC011481 (2024).
11. R. M. Cornell, and U. Schwertmann, "The iron oxides: structure, properties, reactions, occurrences, and uses" in *The iron oxides: structure, properties, reactions, occurrences, and uses* (Weinheim: Wiley-vch, 2003), 2nd edition, pp. 1-664.
12. A. Kereszturi, et al., Analyzing Raman–Infrared spectral correlation in the recently found meteorite Csátalja. Spectrochim. *Acta A. Mol. Biomol. Spectrosc.* **173**, 637-646 (2017).
13. M. P. Reitze, et al., Mid-infrared spectroscopy of alkali feldspar samples for space application. *Mineral. Petrol.* **114**, 453-463 (2020).
14. S. M. Cambier, D. Verreault, G. S. Frankel, Raman investigation of anodic undermining of coated steel during environmental exposure. *Corrosion* **70**, 1219-1229 (2014).
15. S. Li, L. H. Hihara, A micro-Raman spectroscopic study of marine atmospheric corrosion of carbon steel: the effect of akaganeite. *J. Electrochem. Soc.* **162**, C495 (2015).
16. B. Ménez, et al., Abiotic synthesis of amino acids in the recesses of the oceanic lithosphere. *Nature* **564**, 59-63 (2018).
17. A. Steele, J. K. W. Toporski, R. Avci, S. Guidry, D. S. McKay, Time of flight secondary ion mass spectrometry (ToFSIMS) of a number of hopanoids. *Org. Geochem.* **32**, 905-911 (2001).
18. S. Siljeström, et al., Detection of organic biomarkers in crude oils using ToF-SIMS. *Org. Geochem.* **40**, 135-143 (2009).
19. J. Toporski, A. Steele, Characterization of purified biomarker compounds using time of flight-secondary ion mass spectrometry (ToF-SIMS). *Org. Geochem.* **35**, 793-811 (2004).
20. B. H. Stuart, "Infrared Spectroscopy: Fundamentals and Applications" in *Analytical Techniques in the Sciences*, (John Wiley & Sons, 2004) pp. 1-224.
21. G. Socrates, "Infrared and Raman characteristic group frequencies. Tables and charts" in *Infrared and Raman Characteristic Group Frequencies* (Wiley, 2004), 3rd edition, pp. 1-368.
